# Alternatives to antibiotics for sustainable livestock production in the context of the One Health approach: tackling a common foe

**DOI:** 10.3389/fvets.2025.1605215

**Published:** 2025-08-13

**Authors:** Eveline M. Ibeagha-Awemu, Faith A. Omonijo, Laurie C. Piché, Antony T. Vincent

**Affiliations:** ^1^Sherbrooke Research and Development Centre, Agriculture and Agri-Food Canada, Sherbrooke, QC, Canada; ^2^Département des sciences animales, Université Laval, Québec, QC, Canada

**Keywords:** antibiotic growth promoter (AGP), antimicrobial resistance, antimicrobial alternative, AGP-free livestock production, One Health approach

## Abstract

The discovery of the growth promoting effects of antibiotics in the 1940s contributed to the economic efficacy of the livestock industry. In response to increased animal protein demand from the 1950s, antimicrobial use at sub-therapeutic levels for growth promotion and disease prevention (antimicrobial growth promoter, AGP) improved feed-to-weight ratio, meat quality and overall health of livestock. These benefits encouraged the heavy use of AGPs such that about 70% of global use of antimicrobials was for food animals. Despite the numerous benefits of AGPs, the emergence of antimicrobial resistance (AMR) associated with their use and impact on human and livestock health, establishes AMR as a global health plague, affecting man, animal and the environment. Although many countries have banned the use of AGPs in livestock production, efforts to identify effective alternatives have yielded inconsistent findings and only few effective alternative products are currently available. This highlights the need to intensify efforts toward identifying more effective AGP alternatives. While current strategies focus on evaluating the efficacy of single products/class of products that can enhance productivity and health, future strategies should focus on combining multiple approaches. It is also important to acknowledge that no single alternative can fully replicate the same mechanism of action attributed to antimicrobials. This comprehensive review presents recent research findings on AGP use trend before and after bans in many countries, the benefit/mode of action of reported AGP alternatives, the economic impact of AGP alternatives in the context of the One Health approach, the factors militating the search for effective AGP alternatives, research gaps and future action plans for AGP-free animal farm management.

## 1 Introduction

**Figure 1:**
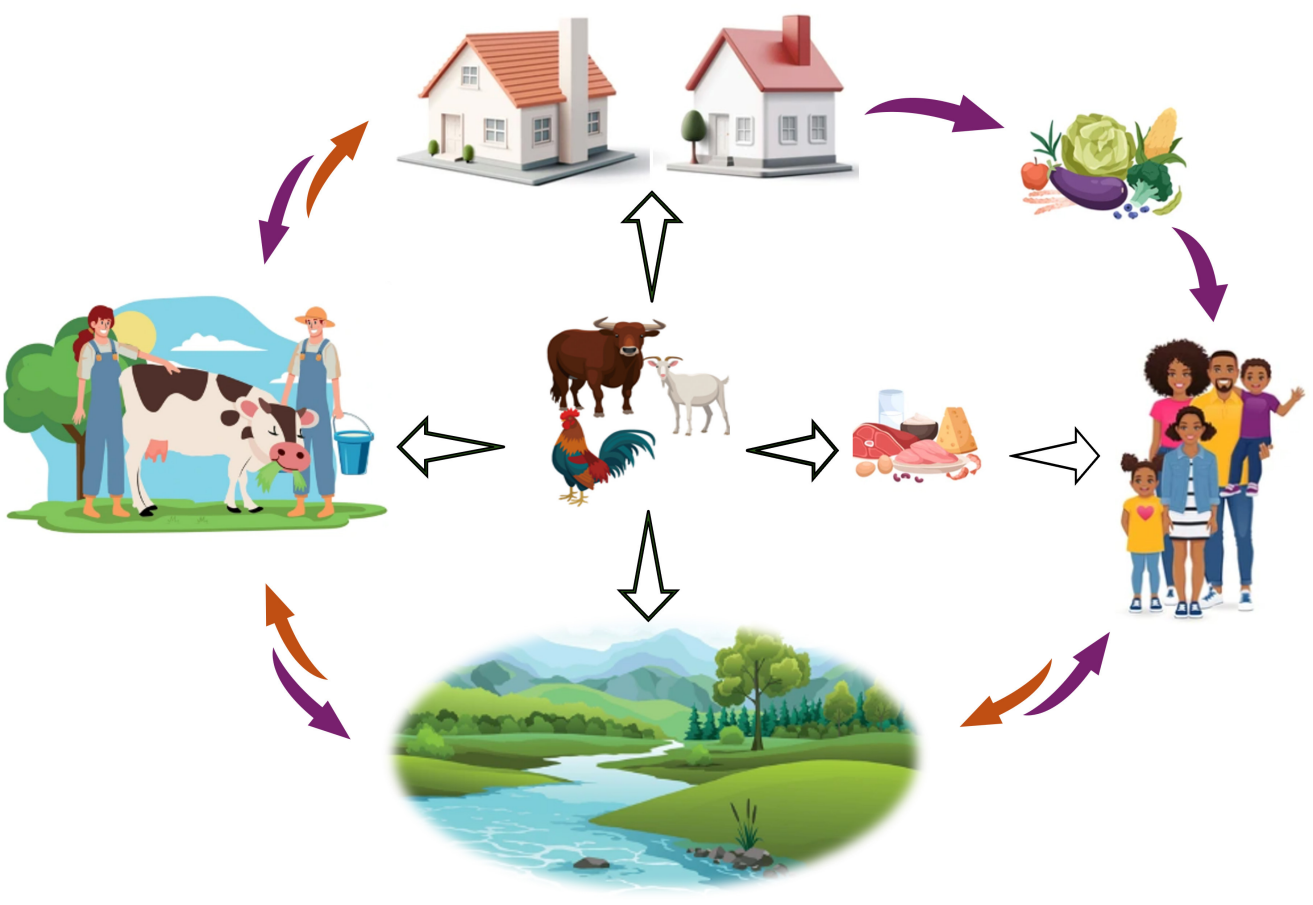
The role of animal farming in the One Health concept of antimicrobial resistance. Farm animals are at the center of the interactions and play a key role in the spread of antibiotic resistance genes and resistant bacteria. Antimicrobial-resistance genes or resistant bacteria of animal origin are transmitted to humans through several routes including the environment, food products and by direct contact with agricultural workers. Animals can directly contaminate fields and water through manure, farm workers, the environment, and food products derived from animals. Contaminated fields and water can also facilitate the transmission to farm workers, as well as to plant and animal products, which ultimately pose a risk to consumers. Finally, the environment can contribute to the contamination of both the farm workers and the general population.

Since the discovery of antibiotics in the 1920s and the subsequent recognition of their growth-promoting effects in the 1940s (1), their use has played a pivotal role in enhancing livestock productivity and in improving the economic efficiency of animal production systems. In response to increased animal protein demand from the 1950s, antimicrobials have been used as feed supplements at sub-therapeutic doses to promote growth by enhancing feed efficiency and preventing disease occurrence in livestock production (2, 3), which ultimately contributed to the intensification of livestock production. Generally, antimicrobials are used in livestock production to (1) treat infected animals (disease treatment or therapy); (2) prevent disease occurrence even in animals that show no visible signs of illness (prophylactic use); (3) prevent the spread of disease to healthy animals in herds where infected animals are present (metaphylactic use or disease control use); and (4) promote faster growth or more efficient livestock growth (use for production purpose which may coincidentally prevent disease occurrence). Since the first use of antimicrobials as feed supplement for the purpose of promoting growth, coupled with advances in genetics, nutrition, management practices, and biosecurity measures, there has been enhanced growth of pigs and poultry, improved feed-to-weight ratio in livestock, improved feed efficiency, meat quality and overall animal health (4–6). These benefits encouraged the heavy non-therapeutic use of antimicrobials or antibiotics for growth promotion and disease prevention (AGPs) such that about 80% of antimicrobial use in the United States was for food animals and over 70% global sale of antimicrobials was for food animals, which is projected to increase by 67% by 2030 in low- and middle-income countries (7–9). Moreover, it was also projected that antimicrobial use in livestock production far exceeds its use for human health purpose (8). While antimicrobials as excellent assassins quickly eliminate their targets (microbes), they also quickly lose their ability to perform effectively as microbes develop defense mechanisms in the form of antimicrobial-resistance genes (ARGs). Antimicrobial-resistance genes circulating in the food chain causes the emergence of antimicrobial resistance (AMR) impacting human, livestock and environmental health (9). Antimicrobial-resistance genes or resistant bacteria of animal origin may be transmitted to humans through several routes including the environment, food products and by direct contact with agricultural workers (8), thus establishing AMR as a global health plague, affecting both man, animal and the environment. Therefore, AMR emanating from antimicrobial use in food animals is an issue within the One Health concept (Figure 1), requiring concerted efforts to address AGP use in livestock production and AMR. Moreover, antibiotic usage in animal feed is increasingly under scrutiny resulting from consumer concerns and demand for products from animals raised without AGPs.

Following the likening of AMR to a “ticking time bomb” in 2013 by Britain's Chief Medical Officer, Sally Davies, who also lobbied for the consideration of AMR as a threat of comparable magnitude to major coastal flooding or catastrophic terrorist attacks (7), there was a deluge of national action plans, expert reports, and pledges by many of the G20 nations and international organizations (World Health Organization (WHO), the Food and Agricultural Organization (FAO), the world Organization for Animal Health (OIE) and individual nations) to reduce antibiotic use (10–14). These pledges and planned actions included funding antibiotic research, tackling human overuse and commitment to reducing antibiotic use (therapeutic and AGPs) in food production. Moreover the guidelines for AMR surveillance established by these organizations allows for clear understanding of how AMR spreads across different settings and specific environments; which allows for the establishment of the relationships between antimicrobial use and AMR in different scenarios (animal, environment and human) and the outcome of interventions within and between the different sectors (11, 14–16).

**Figure 2:**
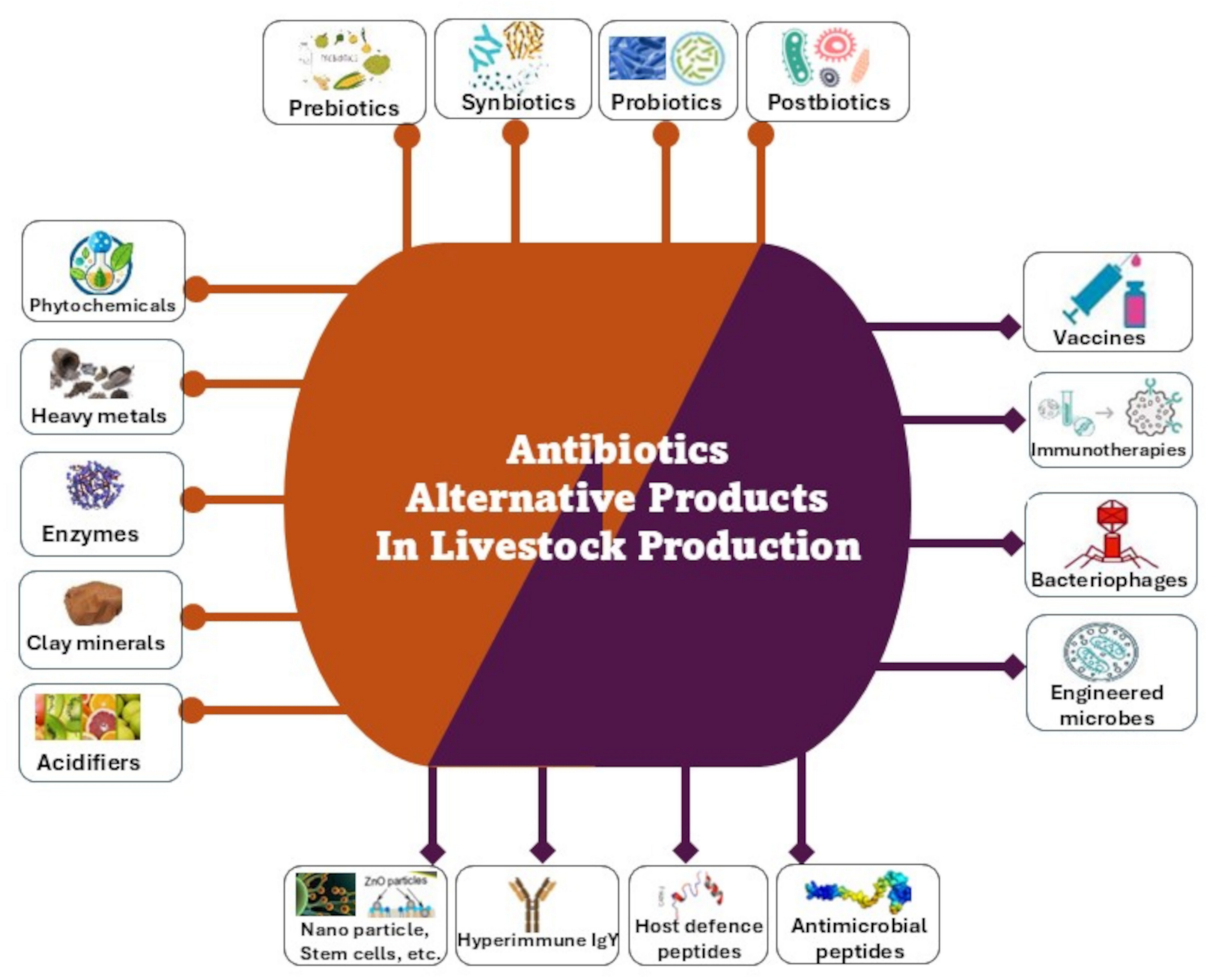
A summary of the health and growth-enhancer alternatives to antibiotics used in animal production or have been experimented. The brown lines point to those alternative products that are mainly administered through food/water. The purple lines points to immunotherapeutic and other microbial compounds or strategies.

As a result of the growing association of AGP use with AMR, many countries have banned/regulated the use of antimicrobials in growth promotion (Table 1) and concerted efforts have been put in place by various countries to develop alternatives to AGP in animal agriculture. An alternative to antibiotics is generally regarded as any substance that can prevent the need for antibiotic use in livestock production or be substituted for antimicrobial drugs (17, 18). Such efforts have seen reports on various AGP alternatives such as microbial related products (probiotics, engineered microbes, bacteriophages, bacteriophage-derived products, etc.), phytogenics (e.g., essential oils, phytonutrients, phytochemicals, acidifiers, trace minerals, etc.), immune-derived products (e.g., antimicrobial peptides, immunomodulators, etc.), prebiotics, vaccines, enzymes, minerals, metals and innovative animal drugs, etc. (Figure 2) and their mode of actions, which are varied and inconsistent in most cases, have been presented in several reviews (6, 19, 20). In spite of the ability of many existing alternatives to AGP to enhance animal production and health, only few effective alternatives to AGP with ability to effectively enhance productivity and prevent/control infections exist necessitating the search for more effective alternatives. This review therefore presents recent research findings on antimicrobial use trends before and after the ban of AGPs by many countries. It also discusses the benefit/mode of action of reported AGP alternatives, the economic impact of AGP alternatives in the context of the One Health approach, the factors militating the search for more effective AGP alternatives, research gaps and future action plan for AGP-free animal farm management.

**Table 1:** Sample regulations and bans on the use of antibiotic growth promoters by various countries.

**Country or region**	**Action**	**Date**	**References**
Sweden	Ban on growth-promoting antibiotics use in all food animals	1986	(331)
Namibia	Ban on hormones and antibiotics use for growth promotion in the beef industry	1991	(33, 332, 333)
European Union (EU)	EU banned use of avoparcin in growth promotion	1997	(334)
	EU banned use of bacitracin, spiramycin, tylosin and virginiamycin in growth promotion	1999	(334)
	EU-wide ban on antibiotics use as growth promoters in animal feed	2006	(3, 335)
	Ban on the importation of meat and dairy products produced with antibiotic growth promoters Fluoroquinolones however continues to be used in the UK and in many EU countries	2022	(336)
USA	Final guidance implementing voluntary plans to phase out the use of medically important antibiotics in livestock for production purposes issued by US Food and Drug Administration (FDA)	2013	(337)
	Ban on the use of medically important antimicrobials in livestock. However antimicrobials classified as medically important by the World Health Organization such as bacitracin and carbadox are still used as AGPs in pig production	2017	(337)
Australia	Antibiotics used in human medicine banned as growth promoters in livestock. Five antibiotics (olaquindox, avilamycin, bambermycin, monensin, and salinomycin) not used in human medicine continue to be used as growth promoters in cattle, sheep, poultry and pigs	2017	(338)
Canada	Sale for veterinary use of medically important antimicrobials (MIAs) by prescription only	2018	(339)
	Growth promotion claims on medically important antimicrobials (MIAs; category I, II, and III antimicrobials) no longer allowed. However, products not considered MIAs, such as ionophore and coccidiostat products, are unaffected	2020	(25, 339)
China	Ban on AGPs use in livestock production. Herbal medicines continue to be used	2020	(340)

## 2 Antibiotics use trend in livestock production before and after AGP bans by many countries

### 2.1 Trends in antimicrobial use in livestock production

Agricultural antibiotics use started with synthetic sulphonamides produced by Bayer (German pharmaceutical manufacturer) and marketed as Prontosil (sulfochrysoidine) in 1935, which was effective against Gram-positive bacterial infections (7). The success of Protonsil and other sulphonamides ushered in a new era of therapeutic drugs, which were marketed for livestock use as early as 1938, prompting the development of different antibiotics products by various United States and European companies for different applications in livestock. For example, gramicidin was used to treat an outbreak of mastitis infection (udder inflammation) in cows at a World Exhibition in New York in 1940 (21). The next decades will witness the growing and expanding use of antibiotics in livestock disease management, and as a production management tool, fueled by increasing demand for animal products. As early as 1948, sulfaquinoxaline (produced by Merk) was officially licensed for routine inclusion in poultry feeds, the so called “medicated feed,” to control coccidiosis. The use of medicated feeds/water helped to curb disease occurrence and spread in concentrated and mostly intensively management animal operations, and enhanced productivity by reducing labor time spent on animal care.

Interest in the non-therapeutic use of antibiotics in livestock production started with observations in 1949 that feeding antibiotic fermentation wastes as a viable alternative source of vitamin B12 feed supplement resulted in increased animal weight gain (22). Moreover, further investigations found that feeding low-dosed AGPs also prophylactically protected against bacterial infections (21, 23). These observations and perceived benefits fueled and expanded the adoption of AGPs in livestock enterprises, which quickly blurred the boundaries between growth promotion, therapy, and prophylaxis on-farm. An in-depth chronicle on the development and application of antimicrobials in livestock production to the emergence of AMR was presented recently (7).

A plethora of evidence abound on the benefits of AGP such as its positive effects on feed efficiency, meat quality, overall growth rate, reproduction performance, egg production and hatchability, etc. (4, 5). For example, it is known that pigs fed AGP require 10%−15% less feed to achieve desired growth (4) with the consequence of reduced cost of production since feed constitute a major expense in animal farming. AGPs ensure enhanced feed to animal product conversion efficiency and overall improvement. Meat from AGP supplemented livestock was of better quality and contained high amounts of proteins than fat compared to non-supplemented counterparts (24). The health of AGP supplemented animals improved while mortality decreased (4, 5). These benefits saw the explosion of the application of AGPs in livestock management for increased productivity and disease control worldwide. Moreover, a plethora of investigations have examined the antimicrobial use trends in many countries (8, 9, 25, 26), and concluded that the most important use of AGP in livestock production in the past 70 decades has been for growth promotion and disease control (prophylactic). Using information on livestock densities, antimicrobial consumption estimates in high-income countries and other variables to estimate antimicrobial use in livestock for years 2010 and 2030, it was found that the mean annual consumption of antimicrobials per kilogram of livestock produced was 148, 45, and 172 mg/kg for chicken, cattle, and pigs, respectively (8). Moreover, the same study estimated that the global consumption of antimicrobials will increase from 63,151 tons in 2010 to 105,596 tons by 2030, which represents a 67% increase (8). Using sales data in 41 countries, global sales of antimicrobials was estimated at 93 thousand tons in 2012 and projected to increase by 11.5% to 104 thousand tons by 2030 (27).

### 2.2 Regulation of antimicrobial use in livestock production

Although antibiotics use as feed additive provides numerous benefits to livestock health and welfare, its widespread use at sub-therapeutic levels poses significant risks to public health due to the development and dissemination of AMR. Moreover, antibiotic residues in animal products by entering the food supply chain is a major threat to human health and the environment (28, 29). These negative consequences have awakened global concerns which regards AMR as one of the greatest public health threats of today (30). To address the growing problem of AMR, many countries issued regulations and bans (Table 1) and action plans (30) aimed at regulating the use of antimicrobials in livestock production and the active search for alternative products (Figure 2). The regulations put in place by many countries have achieved some behavioral change in antimicrobial consumption in some countries. For example, following the ban on the use of medically important antibiotics in livestock production in the United States, there was a significant decrease in marketed volume of antibiotics in 2017, with decrease of 38% in sales volume in 2021 compared to peak sales in 2015, meanwhile annual sales have remained at reduced levels since 2017 (31). However, many developing countries continue unabated use of antimicrobials in livestock production (32, 33), which is not helping with global efforts to curb AMR.

### 2.3 Need to develop alternatives to antibiotics as growth promoters in livestock production

To reduce the over use of antimicrobials to stop the development of new resistant strains, health policy makers worldwide have prioritized tackling AMR because of its profound impact on human, animal, and environmental health (34). In 2015, the WHO published the Global Action Plan on AMR as well as the implementation of relevant strategies (10), emphasizing that elucidation of the mechanisms of AMR in pathogenic organisms from animal farms is essential for the development of new drugs and treatment strategies; and the need to develop alternative products. Finding alternatives to the use of antibiotics in farm animals would obviously have concrete positive consequences for producers, animal welfare, and the various players in the production chain. However, antibiotic resistance is a global phenomenon embedded in most ecosystems and spheres of human activity, and is an interesting example of the One Health concept. The term One Health was developed following the challenges posed by zoonoses and the need to establish collaborations between experts from diverse fields (35). Today, the One Health approach is regarded as a collaborative, multisectoral and multidisciplinary framework that acknowledges the interconnectedness of human, animal, and environmental health. Agriculture, including livestock farming, is a central component of human society and has a great impact on the development and spread of AMR (Figure 1). Overall, antibiotics such as tetracycline for example, are not completely metabolized by animals and therefore end up in the feces (36). Antibiotics in addition to promoting antibiotic resistance in the gut microbiome, may also induce an increase in resistance in the immediate environment. For example, Sun et al. (37) observed that veterinary medicine students experienced modulation of their microbiome, including an increase in ARGs, during a 3-month internship on pig farms. In fact, their microbiome evolved to resemble those of farm workers. Another study by Sun et al. (38) also demonstrated that the microbiome of agricultural workers was different from that of other villagers. Recently, Bai et al. (39) showed that bioaerosols from dairy farms and chickens containing ARGs and antibiotic-resistant bacteria, including *Staphylococcus* spp., can easily spread through the air over distances of up to 10 km. The use of antibiotics in animals is also known to increase the population of antibiotic-resistant bacteria in their feces. The presence of antibiotic residues in feces can be particularly problematic, among other things when feces are used as liquid manure. In fact, as already discussed in other reviews (40, 41), antibiotic residues from animal feces can contaminate plants, vegetables, the environment and people, through the consumption of contaminated products. The overuse of antibiotics in agriculture has sometimes unexpected repercussions. For example, Hammer et al. (42) demonstrated that giving antibiotics to cattle restructures the microbiota and increases the production of methane present in the dung and therefore increases greenhouse gases.

The aftermath of bans on AGP use in livestock production by some countries and the withdrawal of antibiotic use for growth promotion has resulted to increased enteric pathogenic challenges during the early growth phase of many livestock species including poultry and fish, causing immense economic losses in the livestock industry (43). Therefore, decreased AGP use must be matched with the use of products conferring similar benefits as antimicrobials to ensure maintenance of animal productivity, health and welfare, and food security worldwide. Therefore, alternative products that can replicate the effects of antimicrobials in livestock production must be developed. These products can be administered as nutritional additives or as microbial and immune enhancement compounds (Figure 2). The next sections present an overview of some of the progress made in developing alternatives to antimicrobials for use in livestock production.

## 3 Antimicrobial alternatives: microbial and immunotherapeutic strategies

### 3.1 Microbial strategies

A host's microbiome which is made up of microorganisms and their by-products (44) is increasingly recognized as a dynamic microbial organ that can evolve to respond to various factors (45). Livestock microbiomes are essential for health and growth and their dysbiosis has been associated with livestock pathologies such as mastitis (46) and calf diarrhea (47), among others. Moreover, as demonstrated by Verbeek et al. (48), microbial composition and metabolite production correlate with certain aggressive behaviors in pig, such as tail biting. In ruminants where the microbiome is an essential component of digestion, dysbiosis can lead to several pathologies, such as bovine respiratory disease (49), ruminal acidosis (50, 51) and pest des petits ruminants (52).

Antibiotics, being generally broad-spectrum molecules, have repercussions on the composition of the microbiome of animals, even after a single dose, especially if given at the early life (53). Jo et al. (54) demonstrated that finishing pigs treated with lincomycin had a greater incidence of diarrhea and that the animals' microbiota had a greater proportion of detrimental bacteria and fewer bacteria involved in fiber degradation. Currently, it is suggested that a restructuration of the microbiome (including the metabolites produced) and a modulation of the immune response or mitochondria function by antibiotics (55, 56) and other immunotherapeutic strategies (57) could be among the factors promoting animal growth. Although the exact mechanisms still remain hypothetical, the involvement of the microbiome was confirmed in 1963 by Coates et al. (58) who demonstrated that antibiotics had no effect on the growth of chicks without microbiota, compared to birds colonized by a microbial population. Thus, it is increasingly evident that it is necessary to have effective alternatives to antibiotics to treat animals with the hope to see a real reduction in antibiotic resistance. Research in livestock production has explored various methods for improving animal health and performance, including the use of microbiota transplantation (59), engineered microbes and phage therapy.

#### 3.1.1 Microbiota transplantation

Integrating the microbiome as a new variable in animal husbandry for production and health management can reduce the environmental footprint while increasing food yield, and improving animal health. Microbiota transplantation, particularly through fecal microbiota transplantation (FMT), is a part of a broader shift toward understanding and leveraging the microbiome in livestock production. FMT consist of transferring feces from a healthy individual to the gastrointestinal tract (GIT) of another individual presenting symptoms of disease. The feces from the healthy donors contain both health-associated microbes and byproducts of fermentation, including anti-inflammatory compounds, and can resolve symptoms associated with dysbiosis or imbalance of the GIT microbiome. It is a promising method for improving the GIT microbiota in livestock with the potential to improve feed efficiency, alleviate diarrhea, reduce methane emissions, and enhance overall health and performance (60–64). For instance, a study on growing calves found that FMT ameliorated diarrhea and improved growth performance, with specific alterations in the gut microbiota and metabolomic profile correlating with these improvements (62). Similarly, recent reviews have highlighted the potential effect of FMT in pigs, specifically its role in improving GIT microbiota and potential to enhance health and production outcomes (60, 63). These data confirm that FMT can be used as an effective method of preventing or treating GIT disorders as well as improving livestock health and performance.

However, the use of FMT in livestock production also raises biosecurity and regulatory concerns, as the donor's microbiome may contain non-desirable microorganisms, which need to be addressed before widespread adoption (60). Lundberg et al. (65) highlighted the need for systematic experiments to evaluate the stability of microbial transplantations, considering recipient status and housing systems. Canibe et al. (60) discussed the potential application of FMT in pig production, emphasizing the need to identify and optimize factors that can influence its impact. McCormack et al. (66) cautions that FMT may not always lead to the desired outcomes, as seen in a study where FMT from highly feed-efficient pigs had detrimental effects on growth in offspring. These studies collectively underscore the need for further research to fully understand the potential and limitations of FMT in livestock production.

#### 3.1.2 Engineered microbes

Advancement in gene-editing systems such as zinc finger nucleases (ZFNs), transcription activator-like effector nucleases (TALENs), and clustered regularly interspaced short palindromic repeats (CRISPR)/Cas9 have dramatically increased the efficiency of producing gene edited (GE) livestock animals including pig, cattle, sheep and the expansion of the application of GE livestock animals beyond biomedicine (67–69). CRISPR/Cas9 nucleases have shown promise in producing sequence-specific antimicrobials in livestock production, with the potential to target specific bacterial strains and genes (70). Bikard et al. (71) and Citorik et al. (72) both demonstrated the potential of this technology in targeting specific genes in bacteria, such as virulence and antibiotic resistance genes, to selectively kill harmful bacteria. Bikard et al. (71) showed that the development of programmable, sequence-specific antimicrobials using the RNA-guided nuclease Cas9 delivered by a bacteriophage in a mouse skin colonization model had the ability to reprogram Cas9 to selectively kill virulent *Staphylococcus aureus* by targeting virulence genes and to destroy staphylococcal plasmids carrying ARGs (*aph-3* kanamycin resistance gene), while immunizing avirulent staphylococci to prevent the spread of plasmid-borne resistance genes. Other studies further expanded on this by using CRISPR/Cas9 and other systems to induce gene insertion in cattle and other animals to improve health and other traits (73). For example, the insertion of NRAMP1 gene resulted in transgenic cattle with increased resistance to tuberculosis (74). These studies collectively highlight the promising applications of gene editing in developing antimicrobials and enhancing disease resistance in livestock.

However, there are limitations to the use of engineered microbes in livestock production. One primary concern is that CRISPR/Cas9, TALENS and ZFNs can produce off-target mutations in the genome. These unintended alterations can result from the guide RNA inadvertently pairing with unrelated sequences, leading to mutations in genes that are not the intended target (75–77). Various studies have noted that off-target effects could undermine the safety and reliability of CRISPR/Cas9, ZFNs and TALENS applications in livestock (75–77). Although anti-CRISPR proteins have been shown to mitigate off-target activity, yet their utility is limited due to challenges in delivery and biological compatibility (78, 79). Mosaicism is another significant challenge inherent to genome editing in livestock, which can occur post-modification. Mosaicism results from the introduction of edits in only a subset of cells during early embryonic development (80, 81). This genetic variation complicates efforts to produce uniform livestock populations with consistent phenotypes, as not all offspring of a genetically modified parent may express the intended traits (80, 81). Such heterogeneity can pose challenges in breeding programs aimed at enhancing specific desirable traits, limiting the effectiveness of these technologies in producing commercially viable livestock. The public perception of genetically modified animals remains a contentious issue. Concerns regarding animal welfare, environmental impact, and food safety influence consumer acceptance. Therefore, ethical considerations surrounding animal welfare and the potential for unintended consequences from genetic modifications must be addressed to gain public trust and facilitate the adoption of these technologies in production systems (82).

#### 3.1.3 Bacteriophages

Bacteriophages (phages) are ubiquitous in virtually all environments and ecosystems (e.g., soil, ocean, feces, wastewater and the GIT of animals, etc.) (83, 84), and as natural predators of bacteria (85), they play an essential role in regulating bacterial populations (86). Indeed, they represent the most common and abundant biological entities on the planet with an estimated total number of 10^31^ (84, 87), and in some environments they are 10 times more numerous than bacteria (88). As bacteriophages can only infect specific bacteria (89, 90), they are increasingly being investigated as a novel solution to replace antibiotics.

##### 3.1.3.1 Advantages and challenges of phage therapy

Among the existing alternative control methods, bacteriophages are promising as they offer several advantages over antibiotics for the control of bacterial infections and to combat multidrug-resistant bacteria. The existence of several phage-based products approved on the market as antibacterial agents for livestock production, mainly for poultry, but also growing pigs, calves and aquaculture, testifies to the effectiveness of bacteriophages (91). Further advantages associated with phages include their ability to target antibiotic-resistant bacteria and their safety for animals and humans, amongst others (Table 2).

**Table 2:** Advantages and challenges of phage therapy in livestock production.

**Advantages**	**Challenges**
• High specificity: preserve the regular microflora (89, 341) • Ability to evolve since they are biological entities: possibility to outmatch bacterial phage resistance (342) • Environmentally safe biological control method against antimicrobial-resistant pathogenic bacteria (85, 89, 90) • Usage does not add new elements into the environment (85, 342) • No side effects/toxicity even at high doses (89, 116) • Self-limiting (113) • Relatively low cost of discovery (isolation), characterization and production (90)	• High specificity: the target bacteria must be known beforehand (85) • They are not stable because as biological entities, they have the ability to evolve (342) • The need to fully characterize the phages used to ensure their safety (90, 343) • Effectiveness depends on the titer administered (99, 117) • The interactions between phages and the host immune system are still not well-known (94) • Lack of studies and standardized protocols (95)

However, the use of phages in animal production also present challenges, especially for commercial production. Their high specificity requires prior identification of the target bacteria (85), and their efficacy can be influenced by various factors such as temperature, pH, and the physiological status of the host (92). Another challenge in using bacteriophages for livestock production is the feasibility of their administration to animals. Administering phages orally may necessitate antacids due to their sensitivity to low pH (93). Additionally, difficulties in cultivating host bacteria *in vitro* can complicate phage production. Fully characterizing phages through DNA sequencing and genome analysis is essential to ensure they are free of virulence or ARGs (90). The efficacy of phage therapy depends on delivering an appropriate phage titer to the infection site, as an insufficient dose can reduce effectiveness (96, 105, 117). Moreover, interactions between phages and the host immune system are not fully understood (94), and the lack of standardized protocols and regulatory guidelines further complicates their commercial use (95). Table 2 presents a summary of the advantages and the challenges of phage therapy in animal production.

##### 3.1.3.2 Phage administration to livestock

Effective phage therapy requires bacteriophages to reach infection site directly, where the problematic bacteria are located (96–100). Numerous experiments have explored various phage delivery methods to prevent or treat diseases caused by pathogenic bacteria that lead to mortality or reduced performance in livestock, or to reduce GIT colonization by undesirable bacteria for which livestock are major reservoirs (Table 3). These delivery methods include oral gavage, injection, incorporation into feed, milk replacer or drinking water, aerosol spray, environmental dissemination, and *in ovo* injection at the hatchery (in the case of poultry production) (85, 99, 101–110). Keeping in mind that an effective phage therapy depends on whether an important titer of phages reaches the site of infection; the choice of the method used to administer treatment to livestock depends on the type of bacterial pathogen and the stage of infection, as these factors determine the infection site. Moreover, using a combination of administration methods often leads to more effective treatment outcomes. However, it is important to note that some administration methods such as oral gavage and injection are not suitable for some livestock species such as poultry and aquaculture because it would imply the manipulation of thousands of individuals, which is not feasible in a commercial farming context.

**Table 3:** *In vivo* phage trials using various methods of phage administration to livestock.

**Method of phage administration**	**Specie**	**Targeted bacterium**	**References**
Oral gavage	Chicken (broilers)	*Clostridium perfringens* (*C. perfringens*)	(108)
		*Campylobacter jejuni* (*C. jejuni*)	(342)
	Chicken (broiler chicks)	*Campylobacter coli* (*C. coli*)	(85)
		*Salmonella* spp.	(102)
		*Salmonella enterica* subsp. *enterica* serovar Typhimurium (*S*. Typhimurium)	(344)
	Swine (market-weight pigs)	*S*. Typhimurium	(345)
Oral gavage and aerosol spray	Chicken (broilers)	Avian pathogenic *Escherichia coli (E. coli)*	(105)
Injection	Fish	*Aeromonas salmonicida* subsp. *masoucida*	(346)
		*Aeromonas hydrophila*	(101)
		*Plesiomonas shigelloides*	(106)
	Chicken (broilers)	*E. coli*	(98)
Feed incorporation	Swine (grower pigs)	*Salmonella* spp.	(347)
	Swine (piglets)	Enterotoxigenic *E. coli*	(120, 348)
		*Salmonella* spp.	(349)
	Chicken (broilers)	*C. perfringens*	(108)
	Chicken (broiler chicks)	*C. coli*	(85)
Drinking water	Chicken (broilers)	*C. perfringens*	(108)
	Chicken (broiler chicks)	*Salmonella enterica* subsp. *enterica* serovar Enteritidis (*S*. Enteritidis)	(107)
		*C. jejuni*	(350)
Drinking water incorporation and aerosol spray	Chicken (broilers)	Antibiotic-resistant *E. coli*	(105)
Milk replacer incorporation	Cow (dairy calves)	*E. coli* and *Salmonella* spp.	(109)
Aerosol spray	Chicken (broiler chicks)	*E. coli*	(96, 98)
		*S*. Enteritidis	(107)
Environmental dissemination	Cow (calves)	Enterotoxigenic *E. coli*	(110)
	Chicken (broiler chicks)	*E. coli*	(116)

##### 3.1.3.3. Phage selection process for cocktail preparation

Using a phage cocktail, which contains multiple phages targeting different receptors, is preferred over a single phage to prevent the emergence of resistant bacteria (111, 112). Korf et al. (113) found that a phage cocktail was effective in preventing resistance in *Escherichia coli* (*E. coli*), unlike using a single phage. Indeed, by using a phage cocktail, even if the target bacterium acquires resistance to one phage, it is highly likely to remain susceptible to at least one other phage in the cocktail (89, 113).

To control bacterial infections, only virulent phages, which undergo the lytic cycle and avoid DNA exchange with the host, should be used to prevent the spread of resistance or toxin genes (108). Also, the absence of non-desired genes, such as those responsible for AMR, bacterial virulence, and lysogeny, is crucial when selecting phages for a phage cocktail (114). To ensure that the selected phages are well-adapted and effective, it is advisable to isolate phages from the same environment as the target bacteria (115). Phages can be isolated from various environments depending on the target bacteria, including the GIT contents of infected carcasses (85), feces (113), contaminated water (113), wastewater treatment plants (96, 116, 117), slaughterhouse (118), or processing plant (96).

Overall, a good phage cocktail should include phages that specifically infect the target bacterium, are virulent and highly lytic, and can be easily produced with well-characterized host bacteria (113). For instance, Miller and colleagues administered (orally via drinking water, feed or oral gavage) a INT-401 phage cocktail comprising five bacteriophages at 10^5^ PFU/ml to over 900 broiler chickens (0–42 days old) infected with *Clostridium perfringens* (*C. perfringens*) and found that administering INT-401 via their feed or drinking water effectively controlled necrotic enteritis and increased weight gain and feed conversion (108). Moreover, although no optimal lysis yields have been established, phages that replicate quickly and produce a high yield are preferred (119).

##### 3.1.3.4 Bacteriophages as growth promoters

An interesting aspect of using bacteriophages in livestock production is their potential as growth promoters. Indeed, studies have demonstrated that adding phages to feed positively impacts the growth performance of antibiotic-free weaned piglets (120, 121) and grower pigs (122). Additionally, a study showed that supplementing chicken feed with a phage cocktail targeting non-pathogenic *E. coli* significantly improved the feed conversion ratio compared to the control group, and combining this phage cocktail with a commercial probiotic in the feed led to a significant improvement in growth performance (123).

##### 3.1.3.5 Commercially available phage-based products for livestock production

Efforts to use bacteriophages for the fight against pathogenic bacteria has led to the development and emergence of various phage-based products for animal husbandry. Available commercial phage products for animal health have been listed in recent reviews (91, 124). Phage-based products for livestock production are incorporated as food additives in animal feed or drinking water, and some are approved for use in Canada (124). To date, the most targeted bacteria by phage products are *Salmonella* spp., *E. coli* and *C. perfringens* mainly for poultry, but also for swine and calves. A Canadian company (Cytophage Technologies Inc.) has developed AviPhage™, a water-soluble phage solution targeting *Salmonella* and *E. coli* in poultry, which is reported to promote animal growth, improve gut health, lower mortality risks, and reduce undesirable bacterial levels without side effects, while another bacteriophage-based product for bovine mastitis is under active development, but not yet available on the market (125).

The use of bacteriophages is still limited and not generally acceptable because of their potential risks of antibiotic resistance transmission. It was demonstrated recently that ARGs, such as sulfonamide-resistant dihydropteroate synthase (*sul1*), beta-lactamase (*blaTEM*), and erythromycin resistance gene (*ermB*) genes were found in bacteriophage DNA samples, while *ermB* and florfenicol-chloramphenicol resistance gene (*fexA*) were the most common ARGs in the bacterial population (126). Furthermore, limited experiments have been carried out to fully understand their mechanism of action and development of antibiotic resistance, and correct dosage of bacteriophage inclusion in animal diet (127, 128). Therefore, further research is warranted to elucidate the mechanism of action of bacteriophages and their impacts on livestock production.

### 3.2 Immunotherapies

#### 3.2.1 Vaccines

Vaccines stimulate the host's immune system and enable the host to respond effectively to an infection. Vaccines function by presenting antigens to the immune system to generate an adaptive response, among other things through the production of B and T cells (129). They therefore have the considerable advantage, compared to antibiotics, of being passive and thus not require human intervention to make a diagnosis and administer medication. Before B and T cells are activated, the antigens must first be recognized by antigen-presenting cells, which triggers the immune process. Although this process occurs naturally, it is possible to increase its effectiveness by adding immunostimulating compounds, which are known as adjuvants, to the vaccine (130). The adjuvants can be compounds as varied as toll-like receptor agonists, cytokines or saponins. Mineral salts and microparticles also serve to protect the antigen and allow slow diffusion. Recently, polyphosphazenes are also being explored as an adjuvant in vaccines for various animals and could stimulate the innate immune response (131).

Vaccines have been instrumental in effectively controlling the incidence of some livestock pathogenic diseases. For example, *Salmonella* bacteria, a culprit in diarrhea in humans (132) and livestock, is a common pathogen of major food animals such as pig, poultry, and cattle (133). It is particularly worrisome since it can be found at all points of the food chain. In 2017, it caused 95.1 million illnesses worldwide and more than 50,000 deaths (134). In addition, it is considered by the WHO to be a priority two bacterium for antibiotic resistance (135). Fortunately, there are effective vaccines against *Salmonella enterica*. Smith et al. demonstrated in 2018 (136) that the use of a commercially available live attenuated vaccine (Salmoporc) in sows significantly reduced the prevalence of the bacterium in farrow-to-finishing herds. Recently, Schmidt et al. (137) demonstrated that the use of the Salmoporc vaccine induced an increase in cytokine-producing CD4+ T cells specific to *Salmonella* Typhimurium (*S*. Typhimurium) in all organs, with the most marked effects at the level of lamina propria lymphocytes of the jejunum and ileum. In cattle, although vaccines exist, their effectiveness remains limited. A recent study found a partial effect of two commercial vaccines against *Salmonella* Dublin (138). In poultry, vaccination against *S. enterica* is considered effective and reduces the presence of the bacterium at different points in the food chain (139). However, the diversity of *Salmonella* serotypes makes it difficult, if not impossible, to get rid of the bacteria completely on farms. However, new products are regularly developed to increase the effectiveness of vaccine strategies. For example, a new trivalent vaccine (*Salmonella* serovars Enteritidis [O:9, serogroup D], Typhimurium [O:4, serogroup B] and serovar Infantis [O:7, serogroup C1]) has been recently evaluated and shown to reduce the shedding of the bacteria (140). A recent study showed in chicks that taking antibiotic treatment before a live attenuated vaccine against *S*. Enteritidis reduced the effect of the vaccine (141); the vaccine strain being itself a bacterium sensitive to antibiotics. This therefore demonstrates the importance of knowing the parameters that can influence the vaccine effect.

Certain pathogenic strains of the bacterium *E. coli* are also particularly problematic for the food industry and are responsible for several poisonings. Globally, *E. coli* is a bacterium known to be prone to acquiring antibiotic resistance genes and therefore difficult to treat (142). This is the case, for example, of *E. coli* O157:H7 in cattle (143, 144). Weaned piglets are also particularly susceptible to *E. coli* infections, which can result in severe diarrhea. Vangroenweghe and Boone recently showed that the vaccination of piglets with an oral live non-pathogenic *E. coli* F4/F18 made it possible, in addition to offering protection against the bacterium, to increase the zootechnical performance of the animals and to reduce secondary infections by *Streptococcus suis* (145), another problematic pathogenic bacterium for the swine industry. This study illustrates well the interconnection between bacteria sharing the same ecosystem and that vaccines can influence more than one pathogen. Although vaccines exist, handling and associated costs can be prohibitive factors to their use (146, 147). Available vaccines for controlling mastitis in cattle and novel vaccine technologies in veterinary medicine were summarized recently (57, 148).

Noteworthy developments in vaccine technologies in the field of livestock production are undergoing significant transformation, driven by advances in molecular biology, immunology, and veterinary medicine. This evolving landscape highlights a shift from traditional vaccine modalities to more innovative approaches, including mRNA vaccines, viral vector vaccines, and DNA vaccines, which promise enhanced effectiveness against a range of infectious diseases (149–152). For instance, viral vector vaccines such as the ChAdOx1 RVF vaccine have demonstrated significant positive effects. Stedman et al. (152) have reported that this vaccine is not only safe and immunogenic but also offers robust protection across various livestock species against Rift Valley fever, with ongoing field trials supporting its future deployment in veterinary practice. Such advancements highlight the role of innovative vaccine platforms in addressing complex disease challenges in livestock, marking a notable departure from conventional inactivated or live vaccines.

Proper understanding of the roles of genes during infection have been used for the development of messenger RNA (mRNA) therapeutics/vaccines with high specificity, validity and safety for the management of many animal and human diseases, including cancer (153, 154). For instance, a study discovered that vascular endothelial growth factor A (VEGF-A) 165 mRNA transcript enhanced cardiac function when administered after 1 week of post mammary infection in swine (155). Currently, the development of mRNA vaccine has great potential due to their low risk of insertional mutagenesis, low cost of production and high potency. When the vaccine is introduced to the host's cell, the RNA is translated, and the antigen is expressed at the specific site (148). Several mRNA vaccines have entered clinical trials to prevent infectious diseases including influenza virus, Zika and rabies (156). RNA or mRNA vaccine uses part of mRNA that encodes antigens coated in vesicle carriers (148).

Furthermore, DNA vaccination emerges as another promising strategy. Choudhury et al. emphasize how recent developments in genetic engineering have led to the creation of subunit and recombinant vaccines specifically designed for ruminants. These vaccines enhance protective immune responses and have begun to replace traditional methods due to their safety and efficacy (150). Advances in delivery mechanisms and adjuvant formulations are also contributing to the effectiveness of these vaccines, further underscoring the move toward more sophisticated immunization approaches (148, 157).

Emerging research further indicates the necessity for cross-protection strategies, especially given the high variability of pathogens affecting livestock. As Davis et al. (158) stated, the identification of correlates of protection is vital for the rational design of vaccines against highly diverse viral pathogens like African swine fever and foot-and-mouth disease. This information guides the development of vaccines that can elicit cellular and humoral immunity, ideally leading to broader protection across different strains and species. The integration of biotechnological advances into the vaccine development pipeline presents additional opportunities to combat endemic diseases. For instance, irradiated vaccine technologies have been highlighted as solutions tailored to the specific needs of local livestock industries (159). This approach not only addresses disease prevention but also encourages stakeholder participation in vaccination programs, thereby improving overall vaccination coverage and efficacy in target populations (160, 161).

Although vaccines are an important weapon in our arsenal of defense against pathogenic microbes, especially in the context of antibiotic resistance, challenges remain. Vaccine development takes time and money. Also, antigenic variation and economic barriers can impede vaccine adoption (161). Therefore, future directions in research and the regulatory landscape will need to address these challenges to facilitate the successful implementation of new vaccine technologies in livestock health management. Recently, it has been proposed that a “One Health vaccinology” approach, consisting of using an inter-species vaccine strategy, could be a solution, especially for vaccines against zoonoses (162).

#### 3.2.2 Immunoglobulins

Generally, the host first line of defense to infection is known as the innate immune response and is aimed at containing and eliminating the infectious organism from the host system (163). The innate host defenses activate adaptive immune responses by changing cell populations and soluble factors in the affected tissues. The innate host immune responses involve activating the local stroma, release of cytokine and chemokine messengers, attracting and activating neutrophils/heterophils to the affected sites, stimulating macrophages and natural killer cells, stimulating effector molecules like enzymes, collectins, acute phase proteins and host defense peptides, and triggering the complement system (163). Immunoglobulins, also known as antibodies, are effector molecules of humoral immunity and are among the innate immune responders that protect against infectious diseases.

Likewise, colostrum is the first milk produced by mammals after parturition and it consists of a very high level of antibodies (e.g., immunoglobulins) that provide passive immunity to the neonate, thereby protecting them from infectious disease pathogens in the first week of life. Passive immunity can be transferred to susceptible animals using hyperimmune plasma derived from animals with a very high immune response and it is a strategy that can be used to reduce antibiotic application by increasing the immune system's ability to fight infection (163, 164). However, there are several licensed immunoglobulin-based products available for passive immunization of animals, including *E. coli*-specific antibodies for calf; antibacterial bovine serum antibodies for cattle, calf and sheep; clostridial antitoxins for cattle, calf, goat, sheep, swine and horse; tetanus antitoxin for horse, cattle, sheep, swine and goat; anti-West Nile virus antibodies, anti-endotoxin antibodies, antibacterial plasma antibodies and equine plasma for horse; and spray dried plasma for weaned piglet (163). Slaughterhouse plasma, milk, and whey are natural sources of immunoglobulins that are readily available and inexpensive. In an experiment carried out by Hedegaard et al. (165), it was revealed that purified natural plasma IgG obtained from slaughterhouse contained antibody reactivity and possessed antibacterial effects against porcine bacteria, such as *E. coli* O138, *E. coli* F4, *E. coli* F18, and *Salmonella enterica* Diarizonae (165). However, some limitations to the use of immunoglobulins include: (1) purified immunoglobulin products need to be controllable, easy to take orally, and compatible with automatic feeding or drinking systems, (2) formulations should provide optimal shelf life at ambient temperature and resistance to denaturing and fragmentation caused by gut bacteria, (3) most importantly, immunoglobulins need to be certified that they are free of adventitious agents, including porcine circovirus type 2, porcine respiratory and reproductive syndrome virus, and porcine endemic diarrhea virus (163).

#### 3.2.3 Host defense peptides

Host defense peptides (HDPs) have broad spectrum antimicrobial activities against several pathogens, including Gram-negative and Gram-positive bacteria, fungi, parasites and viruses, including multidrug-resistant strains and also regulates immunity, making them valuable in fighting microbial challenges. Several studies have proofed the ability of HDPs (e.g., human cathelicidin LL-37 and human β-defensin-3) in stimulating monocytes and other immune cells to secrete chemokines and cytokines, thus indirectly stimulating the recruitment of immune cells to infection sites (166–168). For instance, Cuperus et al. (166) reported that chicken cathelicidin-2 (CATH-2) displayed potent antibacterial properties while also modulating the immune response in chickens. This dual role enhances not only the immediate defense against pathogens but also the host's overall immune competence, which is particularly advantageous in intensive livestock production systems where stress and disease incidence are high. HDPs can enhance the resilience of the gut microbiota and promote the fitness of the host under pathogenic challenges. This is crucial for animals in high-density farming conditions, where disease outbreaks can significantly impact animal health and productivity (169, 170). A plethoral of investigations have indicated that the immunomodulatory effects of HDPs can stabilize the intestinal microbiota, which is essential for maintaining health and preventing disease outbreaks in livestock (review by Findlay-Greene et al. (171)). Not only that, the molecular characterization of HDPs has opened up avenues for recombinant technologies that can create tailored HDPs for specific pathogens affecting livestock (169). HDPs have low toxicity toward eukaryotic cells making them suitable for applications in livestock, where residue concerns associated with conventional antimicrobials are crucial (172). This characteristic aligns with consumer demands for safer meat products and animal welfare concerns surrounding the use of antibiotics in the livestock industry. Currently, research is ongoing on the possible inclusion of HDPs in animal feed to improve disease resistance and eradicate the use of antibiotics (163, 173). Other strategies include breeding animals with improved innate immunity against different strains of bacteria, including the expression of defense peptides specific to the host. This involves genetically selecting animals based on their ability to produce higher levels of immune peptides that will confer greater disease resistance to future generations.

Despite the advantages of HDPs, their use presents several limitations including stability of HDPs in the GIT of animals. The bioactivity of these peptides can be significantly affected by various conditions such as enzymatic degradation and changes in pH, which may lead to reduced efficacy when administered orally. Studies indicate that HDPs structural integrity can be compromised by the harsh enzymatic environment of the gut, limiting their therapeutic potential (174, 175). Moreover, the need for specific formulation strategies to protect these peptides from degradation represents a significant barrier for their practical application in livestock (175). The high cost associated with the purification and expression of HDPs poses a challenge for their widespread implementation in commercial settings (176). There is a need for scalable production methods that maintain cost-effectiveness in fostering the integration of HDPs into standard livestock management practices (176). Additionally, while HDPs are generally associated with low potential for the development of resistance compared to traditional antibiotics, there is emerging evidence that certain bacterial pathogens can develop mechanisms to evade the actions of these peptides (177). Moreover, the regulatory approval of HDPs for use in livestock can be a lengthy process. There is however a need for the establishment of guidelines on optimal dosing strategies and formulations that ensure the effectiveness and safety of HDPs use in livestock (178).

#### 3.2.4 Antimicrobial peptides

Antimicrobial peptides (AMPs) have recently drawn attention due to their broad-spectrum antimicrobial activities, ability to kill rapidly, less toxicity, cell selectivity and immunomodulatory properties. AMPs are small-sized peptides that are crucial in host immune defense in living organisms, including animals, humans, and plants. Antimicrobial peptides are amphiphilic and positively charged and this feature allows them to bind and penetrate bacterial membrane bilayer to induce pores, causing intracellular leakage. Antimicrobial peptides are produced by most microorganisms and based on biological activity, they can be classified as antibacterial peptides, antifungal peptides, antiviral peptides, antiparasitic peptides, and anticancer peptides (179, 180). These features favors them as potential effective alternatives to antimicrobial use in livestock production. The most common AMPs used in feed supplements are divercin AS7 and microcin J25 (106).

Antimicrobial peptides have been found to improve growth performance, nutrient retention, intestinal histomorphology, and balance GIT microbiota (181, 182) (Table 4). Most AMPs modulate innate and adaptive immune responses by modulating pro- and anti-inflammatory cytokines and chemokines (183, 184). For example, supplementation of broiler feed with microcin J25, protected against several *E. coli* and *Salmonella* strains, promoted growth performance, improved intestinal morphology, reduced the secretion of pro-inflammatory cytokines (IL-1β, TNF-α, IL-6) (184), reduced intestinal inflammation (185), and increased the production of IL-10 and IFN-γ (186). Similar results were obtained when microcin J5 was added to piglet feed (184). The mechanism of action of AMPs is through their antimicrobial activity which is based primarily on the association of positively charged peptides with negatively charged bacterial membrane components (phospholipids and teichoic acids, lipopolysaccharide) that cause permeabilization of bacteria membrane, pore formation and cell lysis (20).

**Table 4:** Advantages and challenges of antimicrobial peptides as alternatives to antibiotic growth promoters.

**Advantages**	**Challenges**
• Interaction of positively charged peptides with negatively charged components of microbial membranes leads to pore formation, membrane permeabilization, and cell lysis after re-localization in the cytosolic membrane (351) • Translocation of specific AMPs into the cytoplasm inhibit main cellular processes (352) • Prevent the expression of pro-inflammatory cytokines in the intestines (353) • Maintain integrity of the intestinal mucosal (353) • Thermostability (354) • Increase growth performance and enhance nutrient digestibility (128) • Modulate gut microbiota and improve intestinal immune functions (128) • Reduce the severity of diarrhea (353)	• Synthesis of chemicals can be expensive (355) • Can be susceptible to oxidation during preparation and distribution of feed (356) • Can be degraded by proteolytic digestive enzymes leading to short half-lives in the intestine (357) • Can react or interact with other feed compounds thereby decreasing their bioavailability (358) • Interactions with other compounds in the feed can change the structure of AMPs and inactivate them (358) • Low production yields (359)

It is important to consider the drawbacks to the use of AMPs. The translocation of AMPs into bacteria cytoplasm may inhibit cellular processes, such as synthesis of DNA and protein (187). There is a risk that bacteria can evolve and become resistant to the use of AMP over time. Increased resistance to some antibiotics was observed in variants resistant to an AMP (e.g., nisin-resistant mutants of *Streptococcus bovis*) (188) (Table 4). Other limitations to the use of AMPs include less stability during feed preparation and storage (183, 189) and lack of proper formulations to improve their bioavailability in the GIT (190).

#### 3.2.5 Hyperimmune egg yolk antibodies

Hyperimmune egg yolk antibody (immunoglobulin Y, IgY) is emerging as a promising immune agent and alternative to antibiotics to combat infectious diseases because it is cost effective for mass production, particularly in livestock. Several egg yolk IgY products are commercially available for disease control in livestock. Egg yolk IgY is mostly administered orally, and it is the most common and convenient route that has been extensively investigated for controlling enteric pathogens (191). Purified egg yolk IgY can also be injected into the animal host via different routes (e.g., intramuscular, subcutaneous, and intraperitoneal) for the control of systemic infections caused by different pathogenic microbes (191).

Studies indicate that addition of IgY to livestock diet serve as preventive, treatment and control measures against pathogenic infections (e.g., *E. coli, S. enterica, Campylobacter* spp*., rotavirus, Cryptosporidium parvum*, and *Eimeria* spp.) as well as enhance host immunity (192, 193). They are less toxic and also environmentally friendly. Egg yolk IgY protect against various infections by preventing pathogen colonization in the GIT, alleviating morbidity or mortality rate and improving growth performance. For example, the induction of passive immunity by oral feeding of hyperimmune egg yolk to young broiler chicks infected with *Eimeria tenella* and *Eimeria maxima* improved body weight gains and enhanced immunity (194).

Despite the promising features of egg yolk IgY as a passive immune agent, the stability of egg yolk antibody IgY in the GIT is very crucial for their effectiveness, which is yet to be clearly elucidated. An *in vitro* study found that the potency of specific IgY was completely lost in a pepsin solution but was mostly retained in trypsin solution, which suggests the degradation of egg yolk IgY in the stomach (195). Likewise, a recent study showed that *in vitro* simulation of static digestion was easily degraded in intestinal phase but not in the gastric phase (196). The contrasting results in these two studies might be due to differences in the composition of the artificial gastric solutions and intestinal solutions. Egg yolk antibody was highly degraded in chicken gizzard (191), thus prompting suggestions for encapsulation for the controlled release and protection of orally administered egg yolk IgY in livestock. To date, the commercial use of IgY products are yet to be approved or endorsed by any regulatory authority (197). Furthermore, high purification is needed for passive immunization of host with IgY via parenteral routes (e.g., intraperitoneal, intramuscular, and subcutaneous routes). The limitations associated with the use of IgY in livestock include: lower stability of IgY due to its higher molecular weight, lower percentage of β-sheet structure and reduced flexibility, decreased activity at pH 3.5 or lower and loss of activity with irreversible change at pH 3 as well as sensitivity to pepsin digestion (198).

#### 3.2.6 Cytokine immunotherapy

Recombinant DNA technology allows the production of large quantities of animal cytokines which can be used as cytokine immunotherapy for the control of livestock disease. Recombinant cytokines can ameliorate the outcome of mastitis infections in cows whose immune systems are impaired by recruiting effector cells into the mammary gland. In addition, they regulate acute inflammatory responses by stimulating phagocytic cell activity. A recent review reported on the therapeutic and prophylactic applications of recombinant interferons (IFNs) in livestock species (199). For instance, Sordillo and colleagues showed that IFN-gamma (γ) reduced the rate of mortality and morbidity caused by endotoxemia from bacterial toxins in cows (200). It was also added that the treatment of cow's mammary gland with IFN-γ before infecting with *E. coli* reduced infection of the udder quarters, reduced clinical scores, and shortened the time of infections when compared with the control (200). Recently, Fan et al. (201) assessed the antiviral effects of recombinant porcine interferons (PoIFN-α and PoIFN-γ) as an emergency treatment for pigs infected with African swine fever virus (ASFV), and showed that administering low doses (105 IU/kg) of recombinant IFNs significantly increased cytokine expression, reduced viral replication, and alleviated clinical symptoms during the early stages of infection. This suggests that recombinant porcine IFNs have high protective antiviral effects against ASFV, providing a new strategy for the prevention of the disease (201). It is however necessary to conduct further research in order to better understand the complex interactions between different pathogens and the host, and how cytokines affect their response. Molecular understanding of the role of cytokines in the host immune system will determine how well they can be used therapeutically in the future.

#### 3.2.7 Others

Further developments already finding applications in human health management include epigenetic immunotherapy, regulatory non-coding RNA immunotherapy, nanoparticles therapy, extracellular vesicles for its role in intercellular communication and stem cell based therapies (202–208). For example, nanoparticles enable the examination and manipulation of genetic material and various types such as nano shells, carbon nanotubes, and gold nanoparticles, hold promise for applications such as diagnosis, treatment delivery, and molecular breeding. It offers tools for enhancing animal health, nutrition, and waste management (209). Antimicrobial nanoparticles have been used to replace antibiotics in extenders, as it prevents bacterial growth without inhibiting sperm viability (210). Nanotechnology holds promise for revolutionizing animal health and livestock production, with applications ranging from disease prevention, food safety and production efficiency to food preservation (211). Stem cell-based therapy presents an opportunity for using cells from livestock to enhance their ability to resist infections without the need for antibiotics application. Mesenchymal stem cells (MSCs) derived from various sources, including bone marrow, adipose tissue, and umbilical cord, have been investigated for their immunomodulatory and tissue repair properties (212). Limited trials in livestock indicate that stem cell-based therapies have potential in treating musculoskeletal disorders (213), reproductive disorders (214), and inflammatory conditions (213). More research in this area is needed to explore the therapeutic potential of MSCs in enhancing livestock health and productivity.

## 4 Antimicrobial alternatives: nutritional strategies

In recent years, several alternatives to AGPs have been explored and those administered to livestock as feed additives (Figure 2), their mode of action, their effects on animal health and productivity, and the advantages and limitations to their use are discussed in the following sections.

### 4.1 Phytochemicals

Phytochemicals, also referred to as phytobiotics, phytogenics, herbals, or botanicals are naturally occurring chemical compounds found in plants or part of plants including herbs and spices, and essential oils that are incorporated into animal feed to enhance animal productivity. Sample research findings on the impact of phytochemical supplements on animal performance are summarized in Table 5.

**Table 5:** Impact of phytochemical feed additives on livestock health and production.

**Livestock**	**Phytochemicals**	**Research findings**
Cattle	*Thymus serpyllum* seed and leaf extracts	A promising feed additive to manage oxidative stress in transition dairy cows (360)
	L-menthol, thymol, eugenol, mint oil and cloves powder	Beneficial effects on ruminal fermentation, reduced inflammation, and modulated the risk of sub-acute ruminal acidosis (SARA) starting from week 3 of supplementation in non-lactating Holstein cows (361)
	Carvacrol, anethole, limonene and fructooligosaccharides	Increased feed intake, no effect on milk production, improved fecal score, alleviated some negative effects of heat stress with no effect on production performance, decreased respiratory rate and thyroxine concentration in mid-lactation Holstein cows (362)
	Menthol, thymol, and eugenol	Increased gut fermentation and production of total volatile fatty acids and butyrate, increased neutral detergent fiber digestibility in dry cows (363)
	Menthol, thymol, and eugenol	Rapid microbial adaptation to diet change, and mitigated adverse effect of high starch diet in non-lactating cows (364)
	*Acacia concinna* and *Saccharum officinarum*	Tend to reduce blood serum biochemistry glucose; tend to increase the concentration of protein, bilirubin, alkaline phosphatase, and antibody in blood serum; tend to increase the concentrations of hematocrit, hemoglobin, and erythrocytes in blood cells of dairy calves (365)
	Carvacrol, cinnamaldehyde, and thymol	Reduced leukocyte counts in the diet of post-weaned dairy calves (366)
Chicken	Menthol, carvacrol, and carvone	Reduced mortality and number of dirty eggs, reduced richness in microbial communities, no effect on the antimicrobial resistance profile or the number of antibiotic resistance genes in commercial layers (367)
	Trans-cinnamaldehyde	Reduced *Salmonella* load in the gut of day of hatch broiler chicks (368)
	Moringa oleifera leaf powder and mulberry leaf powder	Regulated antioxidant status and lipid metabolism by increasing SOD2 and decreasing APOB gene expression in laying hens (369)
	Peppermint (*Mentha piperita*)	Positive effect on feed conversion ratio and egg yolk color at 200 mg/kg dosage rate in laying hens (370)
	Quercetin	Improved laying rate, feed conversion and performance by modulating intestinal environment and liver superoxide dismutase content in laying hens (371)
	*Yucca schidigera* extract (yuccaols, resveratrol)	Improve productive performance, blood profile, and antioxidant enzyme activities in laying hens (372)
	*Panax ginseng* (Saponin glycosides [ginsenosides], essential oils sterols, flavonoids)	No effect on egg production performance of layers, positive effect on egg weight and increase in eggshell which could improve profitability at the start of their laying period in laying hens (373)
	*Allium* spp. (alicin, quercitin, gallic acid)	Improved productivity with no effect on quality and modulated the gut microbiota of laying hens (374)
	Eucommia ulmoides (chlorogenic acid, aucubin, geniposidic acid)	Increased egg production by facilitating nutrient adsorption, reducing inflammation, ameliorating blood lipid, and alleviating insulin resistance in laying hens (375)
	*Baccharis trimera* (Less.) DC leaf extract	Antimicrobial activity against gram negative bacteria (*E. coli* and *Salmonella* spp.) in swine (376)
Swine	Phytogenic feed additive (PFA1, PFA2, PFA3, PFA4, PFA5)^a^	Ameliorated the negative effects of *E. coli* and enhanced growth performance; improved immune response, intestinal morphology, and expression of tight junction proteins in weaned pigs (377)
	Oregano oil, caraway oil, carvacrol, methyl salicylate, and menthol	Provided anti-inflammatory and antioxidant properties by reducing the activity of several immune pathways (NF-kB, interferon α/β, antimicrobial peptide, and collagen pathways) in the small intestine and liver of weaned piglets (378)
	Herbanimals^®^^b^	Improved growth performance when the dietary protein was adequate; improved meat composition (muscle – lean% and reduced muscle – fat%) in protein-restricted nursery pigs (379)
	Cinnamaldehyde	Improved daily weight gain and promotes a softer and less pale meat of finishing pigs (380)
	Menthol, carvacrol, carvone	Potentially increase litter size, and influence gut health of sow and piglets (381)
Swine	FRESTA^®^F^c^	Prevented diarrhea and increased growth performance in weaned piglets (382)
	Herb-All Heat-A^d^, Herb-All Heat-B^e^, and gallic acid	Tend to reduce oxidative damage caused by heat stress and improved performance in primiparous sows (383)
Sheep	Lemongrass leaf extract	Increased preventive effects against oxidative stress, normalized superoxide dismutase levels and increased glutathione levels in sheep red blood cells (384)
	Oregano essential oil (*Lippia graveolens*)	Increased antioxidant activity, shelf life and crude protein content in lamb meat (385)
	Phytogenic rich herbal mixture^f^	Increased feed intake, final body weight and dressing percentage; decreased final blood and liver tissue malondialdehyde concentration in heat stressed lambs (386)
	Neem leaf powder	Increased body weight gain (387)
	Mesquite extract (*Prosopis juliflora*)	Increased digestibility, nitrogen balance, final body weight, and performance in sheep raised on pasture (388)
	Mesquite pods (*Prosopis juliflora*)	Improved nutrient intake, nutrient digestibility, carcass weight, animal performance, commercial cuts, external carcass measurements, leg weight, and femoral muscles in sheep finished on rangeland (388)
	Mesquite piperidine alkaloids (*Prosopis juliflora*)	Decreased enteric methane emission in lambs (389)
Goat	Ginger (*Zingiber officinale*), garlic (*Allium sativum*), Artemisia (*Artemisia vulgaris*), and turmeric (*Curcuma longa*)	Increased the relative abundances of phylum Bacteroidota, Proteobacteria, Prevotella, and Rikenellaceae RC9 Candidatus Methanomethylophilus, but decreased Firmicutes, Fibrobacterota, Ruminococcaceae and the archaeal genus Methanobrevibacter in the gut of male goats (390)
	*Boswellia sacra* resin	Decreased total short-chain fatty acids and branched-chain volatile fatty acids concentrations; decreased blood plasma glucose, nonesterified free fatty acid, and β-hydroxybutyrate concentrations; increased milk yield, energy-corrected milk, net energy for lactation, and feed efficiency; and decreased milk somatic cell count (391)
	Bitter leaf *(Vernonia amygdalina)*	Increased feed intake and weight gain, and reduced fecal worm egg count of West African Dwarf goats (392)
	Four phytogenic plant leaf meals (1. *Mangifera indica*, 2. *Nauclea latifolia*, 3. *Gmelina arborea*, and 4. *Alchonea cordifolia*)	Improved performance, nutrient intake, body weight gain, feed conversion ratio, nutrient digestibility and use of West African Dwarf goats (393)
	Soursop leaf meal (*Annona muricata*)	Increased body weight gain, dry matter intake, hemopoiesis and health status (394)
	Scent leaf (*Ocimum gratissimum*)	Increased red blood cells, packed cell volume and hemoglobin; decreased serum enzymes, urea and cholesterol levels of West African Dwarf goats (395)
	Lippia alba hay	Increased daily intake of dry matter and acid detergent fiber, milk yield, and solids content, with no negative effects on rumen and hematological parameters of Alpine goats (396)

^a^PFA1—bitter citrus extract, PFA2—microencapsulated blend of thymol and carvacrol, PFA3—mixture of bitter citrus extract, thymol, and carvacrol, PFA4—premixture of grape seed, grape marc extract, green tea, and hops, and PFA5—fenugreek seed powder.

^b^Herbanimals^®^ (*Pandanus amaryllifolius* Roxb, *Phyllanthus niruri, Amomum cardamomum, Zingiber zerumbet, Apium Graveolens, Anethum Graveolens, Ocimum americanum, Cinnamomum burmannii* Blume, *Myristica fragrans* Houtt, and *Zingiber officinale roscoe*).

^c^FRESTA^®^F (caraway oil [d-carvone], lemon [limonene], dried herbs and spices [clove powder, cinnamon powder, nutmeg powder, onion powder, pimento powder, orange peel powder, peppermint powder and chamomile powder]).

^d^Herb-All Heat-A (*Emblica officinalis, Foeniculum vulgare, Citrus sinensis* and nut fiber).

^e^Herb-All Heat-B (*Andrographis paniculate; Glycyrrhizia glabra; Tinospora cordifolia*; and nut fiber).

^f^Phytogenic rich herbal mixture (Rosemary leaves [*Rosemarinus officinalis*], cinnamon bark [*Cinnamomum zeylanicum*], turmeric roots [*Curcuma longa*], and clove buds [*Eugenia caryophyllata* Thunb]).

#### 4.1.1 Herbs and spices

Herbs and spices of different types (e.g., thyme, oregano, rosemary, marjoram, yarrow, garlic, ginger, green tea, black cumin, coriander, and cinnamon) have been used in poultry, swine, beef, and dairy production, for their potential as alternatives to AGPs (215, 216).

A range of studies have explored the potential of herbs and spices as alternatives to antibiotics in livestock production (217, 218) (Table 5). Thyme, turmeric, garlic, and cinnamon have all been shown to have growth-promoting effects and antimicrobial properties (218, 219). For instance, Seidavi et al. (218) and Díaz-Sánchez et al. highlighted the potential of various spices (thyme, coriander, and turmeric) in improving poultry health and performance. Turmeric has been found to improve growth performance, gut health, and short-chain fatty acid production in weaned piglets (220). In a recent study, administration of cinnamon and turmeric powder as antibiotic growth promoter substitutes in broiler chickens resulted to increased body weight gain, higher feed intake and feed conversion ratio, improved hematological parameters, and increased ileal and cecal *Lactobacillus* populations while decreasing coliforms and *E. coli* (219). *In ovo* supplementation of herbal compounds, such as grape pomace, resveratrol and chicoric acid, enhanced hatchability, chick quality, and post-hatch performance, while reducing infections and oxidative stress (221, 222). These natural alternatives can help improve poultry performance and reduce the risk of antibiotic-resistant pathogens.

Although some herbs and spices have shown potential as alternatives to AGPs, there are still some limitations to their use such as the requirement of optimal dosage to achieve desired health effects; highly lipophilic nature which limits delivery to enteric pathogens; offensive odor; inconsistent results due to variations in product compositions; can be toxic at high dosage; and high cost of production (19, 223).

#### 4.1.2 Essential oils

Essential oils are becoming increasingly popular as potential antibiotic alternatives for animal production based on their antibacterial, antimicrobial, anti-inflammatory, and antioxidative properties, and ability to enhance feed palatability and gut health (19, 224). Essential oils from thymol, carvacrol, cinnamaldehyde, clove, coriander, star anise, ginger, garlic, rosemary, turmeric, basil, caraway, lemon, and sage are generally recognized as safe for their intended use, and they have been used either individually or synergistically to improve animal health and performance (215, 225).

The addition of essential oils to the diets of livestock has been shown to improve production performance (226) (Table 5). The inclusion of essential oils in the diet have been shown to alter and stabilize intestinal microflora and decrease toxic microbial metabolites in the GIT of animals (225). For instance, the synergistic use of carvacrol and thymol in broiler's feed increased their growth performance, enhanced the activities of digestive enzymes, antioxidant enzymes, composition of fatty acids, and immune responses (227). Essential oils could also exhibit their mode of action via immunomodulatory effects including increased production of immune cells and antibodies, and significant expression of cytokines. For instance, thymol attenuated lipopolysaccharide-induced inflammation in intestinal porcine enterocytes (IPEC-J2 cells) by blocking the production of reactive oxidative species, reduced the expression of IL-8 and TNF- α, reduced cell permeability and transepithelial electrical resistance (215). Supplementation of cinnamaldehyde to lactating Holstein dairy cattle diet improved nitrogen metabolism, reduced milk somatic cell count and increased efficiency of feed use (228).

While a plethora of investigations have documented positive impact of essential oils on animal growth and health, factors such as bad odors, inconsistent results due to variations in product compositions, high volatility, need of high doses to obtain desired results, toxicity and high cost of production limit their application (19, 20).

### 4.2 Probiotics

Probiotics, also known as direct fed microbials, are live microbial feed supplements which beneficially affect the host by improving intestinal microbial balance. Several species of bacteria (*Bacillus, Bifidobacterium, Enterococcus, Lactobacillus, Streptococcus, and Lactococcus* spp.; and *yeast* [*Saccharomyces* spp.]) have been used as probiotics in livestock feeding (poultry, dairy, swine, and pig) (229, 230).

Probiotics have been shown to improve growth performance and reduce pathogenic bacteria in animal GIT (229). A plethora of investigations have shown that probiotics, alone or combinations of different beneficial bacteria and/or yeast have growth-promoting effects on poultry (500), swine (20) and ruminants (231, 232) (Table 6). The mode of action by which probiotics exhibit their beneficial effect was recently reviewed (231, 232). Probiotics modulate gut microflora by competing with pathogenic bacteria for nutrients; favor the growth of beneficial bacteria against pathogenic bacteria; production of lactic acid and short chain fatty acids, and reduction of pH to create a hostile environment for pathogenic bacteria; secretion of antibacterial substances like bacteriocins; detoxification of inhibitory chemicals like amines or nitrates, and prevention of bacterial adherence and translocation (233). Furthermore, probiotics also function to maintain epithelial cell homeostasis and survival via the production of cytokines, prevent cell apoptosis and improve cell regeneration, improve barrier function by modulating cytoskeletal and epithelial tight junction, and increase synthesis of mucin (234, 235). Probiotics regulate host immune responses to pathogenic bacteria by reducing pro-inflammatory cytokines; increasing production of IgA; and promoting specific and non-specific immune responses against pathogens (236). Sample data on the impact of probiotics supplemental feeding on livestock health and production are summarized in Table 6.

**Table 6:** Impact of probiotics feed additives on livestock production and health.

**Livestock**	**Probiotics**	**Research findings**
Cattle	*Escherichia coli* Nissle 1917	Significant decrease in methane production in the rumen of cows (397)
	*Lactobacillus plantarum* GB-LP1	Decreased relative abundance of pathogenic bacteria, decreased ammonia nitrogen (NH_3_-N) concentration without altering pH and lactic acid concentration in lactating cows (398)
	*Enterococcus faecium*	Decreased the abundance of erythromycin-resistant enterococci in Holstein steers (399)
	*Saccharomyces cerevisiae* and *Megasphaera elsdenii*	Reduction in NH_3_-N concentration in beef cattle finishing cows (400)
	*Bacillus licheniformis* and *Bacillus subtilis*	Improve nutrient digestibility and utilization in Holstein steers (401)
	Genetically engineered *Bacillus pumilus* strain	Increased inhibition against pathogens growth in beef cattle (402)
	Commercial direct fed microbial (DFM) product^a^	Reduced fecal shedding *E. coli* O157 in finishing cattle in a commercial feedlot (402)
	(CRL2074, CRL2085 and CRL2069)^b^	Increased the abundance of beneficial bacteria (Firmicutes, Actinobacteria), reduced the abundance of Bacteroidetes, increased digestion in steers (403)
	Yeast products (live yeast, yeast cell wall, yeast culture)	Improved rumen health and increased nutrient digestibility of beef cattle (404)
	*Saccharomyces cerevisiae* and *Lactobacillus acidophilus*	Improved feeding and milk yield, reduction of pathogens in the gut, and enhanced mucosal immunity in Holstein-Friesian cows (405)
	Multi-species probiotics^c^	Improved productive and reproductive performance of postpartum lactating cows (406)
	*Saccharomyces cerevisiae* CNCM I-1077	Improved body weight, average daily gain and gain:feed ratio on d 47 in receiving and backgrounding steers (407)
	*Saccharomyces cerevisiae*	Increased carcass quality with increasing yeast inclusion; increased total tract apparent digestibility of dry matter, organic matter, crude protein ether extract and fiber in finishing cows (408)
	*Saccharomyces cerevisiae* Sc47 CNCM I-4407	Increased digestibility of dry matter and fiber in beef cattle (409)
	*Saccharomyces cerevisiae*	Improved average daily gain and ruminal propionate concentration; effect on rumen microbial composition by increasing the abundance of Ruminococcacea in fattening beef cattle (410)
	*Saccharomyces cerevisiae* CNCM I-4407	Increased milk yield, total volatile fatty acid (VFA) and acetate concentration; reduced ruminal lactate, serum non-esterified fatty acids (NEFA) and beta-hydroxybutyrate (BHBA), and liver enzyme activities in early lactation dairy cows (411)
	*Megasphaera elsdenii*	Reduced *Streptococcus bovis* count, increased protozoa count in ruminal cannulated heifers (412)
	*Saccharomyces cerevisiae* and *Lactococcal*	Alleviated mastitis infection by relieving inflammation of the mammary gland, reduced milk somatic cell counts, reduced abundance of mastitis pathogens (*Enterococcus* and *Streptococcus*) in lactating dairy cows (413)
Sheep	Commercial zootechnical additive DBR SACCH^®^ Probiotic Concentrate	Increased silage and dry matter intake. Increased abundance of Azoarcus and Dialister taxa in the rumen fluid and Treponema and Fibrobacter taxa in the fecal microbiome of lambs (414)
	*Saccharomyces cerevisiae*	Reduced expression of pro-inflammatory genes, increased milk yield; enhanced energy utilization in dairy sheep (415)
	*Lactobacillus casei, Lactobacillus plantarum*	Improve meat tenderness and flavor in lambs (416)
	*Bacillus licheniformis, Bacillus subtilis* and *L. plantarum*	Increased muscle fibers density, increased intramuscular fat deposition and improved meat quality in lambs (417)
	*Bacillus subtilis, Bacillus licheniformis*	Improved digestibility, growth performance and blood metabolites of fattening lambs (418)
	Grow K Probio	Improved average daily weight gain, daily dry matter and total digestible nutrient (TDN), increased nutrient digestibility, decreased rumen NH_3_-N, increased total volatile fatty acids, improved feed conversion and economic efficiency of growing lambs (419)
	*Ruminococcus flavefaciens* (powder or liquid)	Increased *in vitro* dry matter digestibility, improved lambs daily gain and nutrient digestibility in growing lambs (420)
Goat	Commercial product (Goats Prefer^®^ Probiotic Plus Paste, Vets Plus, Inc, Menomonie, WI, USA)	Improved innate immune markers, including total protein and IgA levels, particularly during the pre-weaning period (1 month post-supplementation). Reduced the prevalence of *E. coli* virulence genes in pre-weaned goats in pre and peri-weaned goats (421)
	Autochthonous strain *(Limosilactobacillus mucosae CNPC007)*	Improved the nutritional, functional, aromatic, and sensory properties of goat ricotta cream (422)
	*Limosilactobacillus fermentum WXZ 2-1*	Improved the texture characteristics, antioxidant capacity, and flavor of fermented goat milk (423)
Swine	*Bacillus strains*-Provent ECL (lot CX0020180321)	Modulated innate immunity to fight infections in the respiratory tract, reduced the rate of lung colonization by *Salmonella enterica*, decreased the presence of porcine reproductive and respiratory syndrome in the lung, and reduced the severity of gross lung pathology in piglets (424)
	*Bacillus subtilis*	Increased villi height in duodenum and ileum, and villi height:crypt depth in all the segments of small intestine. Increased weight gain and improved feed:gain ratio in heat stressed pigs (425)
	Multi-strain *Bacillus subtilis*	Improved growth performance, amino acid and nitrogen digestibility in weaned pigs (426)
	RC016 and VM004^d^	Decreased diarrhea, stomach ulcers, respiratory signs; increased carcass weight; significantly reduced lumbar fat thickness; improved performance of weaned piglets (427)
	*Lactobacillus plantarum*	Inhibited the growth of pathogenic bacteria (*E. coli, S*. Typhimurium and *Staphylococcus epidermidis*), resistance to box-bile salt and low pH 3.0; ability to produce high levels of folate and riboflavin in swine farming (428)
	*Bacillus subtilis* bacterium (CP9)	Attenuated enterotoxic *E. coli* induced cytotoxicity in IPEC-J2 cells, inflammatory response by reducing nitric oxide production and expression of the Toll-like receptors; reinstated cell proliferation and increased relative expression of tight junction genes (claudin-1, occludin, and zona occludens-1) in IPEC-J2 cells derived from jejuna of neonatal piglets (429)
	*Acidilactici* FT28 probiotic	Reduced mortality in suckling piglets by improving systemic immunity and intestinal integrity in suckling piglets (430)
	*Enterococcus faecium*	Reduced oxidative stress and paracellular permeability of IPEC-J2 cells; inhibited the adhesion of *S*. Typhimurium and *E. coli* in IPEC-J2 cells (431)
	*Lactobacillus johnsonii* BS15	Increased daily gain and feed conversion rate, improved intestinal development and digestion of piglets (432)
	*Lactobacillus reuteri* and *Lactobacillus salivarius*	Improved the composition of intestinal bacteria which resulted to improve growth performance of weaned piglets (433)
Chicken	*Bacillus subtilis*	Increased breast muscle total antioxidant capacity and improved meat quality (tenderness, water holding capacity, tastier) of broiler chickens (434)
	*Lactobacillus acidophilus* D2/CSL	Increased the height of the mucosal in layer hens and enhanced production performance in rurally reared chicken (435)
	*Lactobacillus plantarum* 16	Promoted the expression of interferon-γ, IL-6 and IL-10 in the ileal mucosa, and reduced the expression of Cox2 alkaline phosphatase and creatine kinase in broiler chickens (436)
	*Lactobacillus plantarum* JM113	Enhanced the digestion, absorption, and metabolic functions of the gut and increased the abundance of beneficial bacteria. Decreased apoptosis and intestinal inflammation induced by deoxynivalenol in broiler chickens (437)
	*Bacillus subtilis*	Improved growth performance, particularly when the probiotic was delivered in-feed or in-water. Positive effects on the morphology of the intestinal lining and cecal short-chain fatty acid concentration, suggesting increased gut health in broiler chickens (438)
	*Lactobacillus kefiranofaciens* DN1	Prevent the colonization of *S*. Enteritidis in chicken gut (439)
Horse	PROBIOPlus^^®^ e^	Decreased fecal pH and increased bacterial populations of the Ruminococcaceae family which could maintain healthy gut microbiome during antibiotic treatment that could positively impact the gut microbiota of standardbred horses (440)
	*Bacillus coagulans* GBI-30, 6086	Reduced production of inflammatory genes (SAA, IL-6, and PGE_2_) and ameliorated the postexercise inflammatory response in horses (441)
	ProVetin^f^	Increased foals birth weight and growth rate (442)

^a^Commercial direct fed microbial (DFM) product (lactic acid and propionic acid bacteria).

^b^CRL2074 (*Lactobacillus acidophilus*), CRL2085 (*Limosilactobacillus fermentum*) and CRL2069 (*Limosilactobacillus mucosae*).

^c^Multi-species probiotics (*Saccharomyces cerevisiae, Bacillus subtilis, Enterococcus faecium, Lactobacillus acidophilus, Bifidobacterium tedium*).

^d^RC016 (*Saccharomyces cerevisiae*) and VM004 (*Kluyveromyces marxianus*).

^e^PROBIOPlus^®^ (Ruminococcaceae family).

^f^ProVetin (*Bacillus subtilis, Saccharomyces cerevisiae, Trichoderma reesei*).

Despite the promising effects of probiotics as alternatives to AGPs; limitations to their use abound, such as differences in the quality and dose of some probiotic products, poor rate of survival in the GIT, inactivation of live beneficial bacteria during feed manufacturing, storage or transport, and allergy and transmission of antibiotic resistance genes (20, 237).

### 4.3 Prebiotics

Prebiotics are non-digestible feed components (non-starch polysaccharides or oligosaccharides such as mannan-oligosaccharide; fructans [fructooligosaccharide and inulin], galactans [galacto-oligosaccharide], malto-oligosaccharide, lactulose, lactitol, and gluco-oligosaccharides) and so on that stimulate the growth of beneficial bacteria in the GIT (238, 239). These non-digestible oligosaccharides are fermented in the GIT by beneficial bacteria and serve as a source of energy for the microbiota.

Prebiotics administration have shown promise in improving growth, immunity, and intestinal health in animals. Prebiotics perform several functions, including inhibition of pathogen attachment to cells, immunomodulation, increased the concentration of antibodies (IgG and IgM), alteration of gut morphology, reduction of intestinal pH, impact microbial populations (e.g., increase population of beneficial. bacterial strains such as *Bifidobacterium* spp. and *Lactobacillus* spp.) and decrease pathogenic bacteria (e.g., *E. coli* and *Salmonella* spp.), and promote fermentation-based synthesis of antimicrobial chemicals (20, 240). Prebiotics have also been shown to abate heat stress (241); and improve meat quality traits of chicken without affecting lower redness index, lightness and yellowness (242). A recent review on the prebiotic effects of seaweed polysaccharides demonstrated that they may be used to promote pig health throughout the production cycle, hence lowering antibiotic use (243). Sample studies showing the impact of prebiotics on livestock health and production are summarized in Table 7.

**Table 7:** Impact of prebiotics feed additives on livestock health and production.

**Livestock**	**Ingredients**	**Research findings**
Cattle	RumiForm Digest^a^	Increased weight gain in dairy calves (443)
	Calcium carbonate, alfalfa meal, wheat middings, extract of *Yucca schidigera* and yeasts.	Reduced the prevalence of *E. coli* in beef calves (444)
	Vegetable pellet feed (mannan oligosaccharides, β-glucan oligosaccharides, purified lignin)	Improved production and several hematological items of early lactation dairy cows; linear increase in milk yield and milk protein percentage; decreased milk urea nitrogen (445)
	Commercial mannan-oligosaccharides	Improved growth performance, health scores and serum biomarkers in male calves (446)
	Celmanax™	Acted as an anti-adhesive for enterohemorrhagic *E. coli* colonization and a mycotoxin binder in *in vitro* studies (447)
Swine	Laminarin and fucoidan (*Laminaria* spp.)	Laminarin increased body weight and decreased IL-6 and IL-8 mRNA expression in the colon and ileum, respectively. Fucoidan increased body weight in grower-finisher pigs (448)
	Laminarin and fucoidan (*Laminaria* spp.)	Improved feed efficiency, fecal scores and volatile fatty acid production in the colon. Reduced fecal counts of *S*. Typhimurium and gene expression of IL-22 in the colon in grower-finisher pigs (449)
	Laminarin (*Laminaria* spp.)	Increased average daily gain in newly weaned pigs (450)
	Laminarin and fucoidan (*Laminaria* spp.)	Improved performance and prevented post-weaning intestinal dysfunction in piglets (451)
	Seaweed extracts (laminarin, ash and fucoidan)	Improved average daily gain and gut health of weaned pigs challenged with fecal enterotoxigenic *E. coli* (448)
	Laminarin rich macroalgal extract	Improved piglet performance after weaning by promoting the proliferation of bacterial taxa such as *Prevotella* that could enhance nutrient digestion while potentially reducing pathogenic bacterial taxa counts, such as Enterobacteriaceae (452)
Goats	Oligosaccharides (fructooligosaccharide and galactooligosaccharide)	Increased the growth of beneficial bacteria (Bifidobacterium and Ruminococcus-gnavus) in the gut and improved the metabolism of short-chain fatty acid in fermented goat milk (453)
	Pectin oligosaccharides	Prevented adhesion of uropathogenic microorganisms, and protected colonic cells against Shiga toxins in newborn goats (454)
	Inulin (fructooligosaccharide)	Improved carcass yield and blood parameters and no effect on meat quality of goat kids (455)
	Sangrovit^®^ (*Macleaya cordata*, Phytobiotics Futterzusatzstoffe GmbH, Austria)	Reduced oxidative damage in the small intestine in young goats lower gut (456)
Chicken	Commercial product (Fortibac^®^)	Increased the relative abundance of phylum Bacteroidetes, family Ruminococcaceae, and family Lachnospiraceae. Increased feed conversion ratio in broiler chickens (457)
	Trans-galactooligosaccharides; extract of *Laminaria* spp., raffinose family oligosaccharides	Increased growth performance, carcass and meat quality traits in broiler chickens (458)

^a^RumiForm Digest1 (yeast, sorbitol, sodium chloride).

Despite the benefits of prebiotics, its administration in large quantities might cause bloating or diarrhea (20, 244), and their effectiveness can be inconsistent across different animal species or environmental conditions (245, 246). Furthermore, competition from pathogenic organisms also limits the utility of prebiotics. Some pathogens can utilize prebiotics for their benefit, leading to increased resistance or growth rates of harmful bacteria. Fuhrmann et al. (246) reported that both single applications of the prebiotics (Inulin and fructooligosaccharides) failed to significantly reduce *E. coli* fitness. This suggests that adverse interactions can occur when prebiotics are included in certain dietary contexts, underscoring the complexity of gut microbiota interactions and the necessity for additional research to optimize prebiotic applications (247). Prebiotics also have the potential to develop resistance such as co- and cross-resistance with antibiotics (248).

### 4.4 Synbiotics

Synbiotics refers to the synergistic use of probiotics and prebiotics to counteract the challenges faced with the survival of probiotics in the intestines. The combined use of probiotics and prebiotics in livestock is reported to be more efficacious on gut microbiota (240), as it increase the production of short chain fatty acids and lactic acid, reduce the concentrations of branched chain fatty acids (20, 240), and increase digestibility and daily weight gain (501). For instance, lambs fed with *Saccharomyces cerevisiae* and mannan oligosaccharide had improved growth performance and daily weight gain with little effects on carcass traits and visceral mass (249). Sharma et al. (250) investigated the effect of synbiotics (fructo-oligosaccharide, and *Lactobacillus plantarum* CRD-7) on the growth performance, nutrient digestibility and fecal microflora in murrah bufallo calves and reported increased digestibility, average daily gain, immune response, antioxidant enzymes, fecal microbiota and metabolites, and reduced fecal score and incidence of diarrhea.

The mechanisms by which synbiotics affect the host include the use of prebiotic to promote the growth of probiotic bacteria in the intestines. Synbiotics impact the immune system of the host via the production and maturation of leukocytes. A study in broiler chickens showed that early *in ovo* treatment with synbiotics modulated the production and maturation of leukocytes. Likewise, the combined use of the *Bifidobacterium* breve probiotic and galacto-oligosaccharides prebiotic significantly enhanced defense against fatal intestinal infections due to multidrug-resistant *Acinetobacter baumannii* in a mouse model (251). Recent data on the impact of synbiotics on livestock production and health is summarized in Table 8.

**Table 8:** Impact of synbiotic feed additives on livestock health and production.

**Livestock**	**Ingredients**	**Research findings**
Cattle	Yeast cell wall and commercial mannan-oligosaccharides	Improved the growth performance and health status of neonatal calves (446)
	Lactobacillus plantarum and inulin, oligofructose, β-glucan, and polydextrose	Reduced aflatoxin B_1_ and its bioaccessibility in artificially contaminated ultra-high temperature cow milk (459)
	Symbioveba^^®^ a^	Decreased the prevalence of *Staphylococcus aureus* and *E. coli* in early lactation dairy cows (460)
	Synbiotic supplement (yeast-derived prebiotic + *Bacillus subtilis* probiotic)	Plasma glucose concentrations were increased; improved response to respiratory disease treatment in beef steers (461)
	Yeast-derived prebiotic + *Bacillus subtilis* probiotic	Increased feed intake; high marbling score; larger longissimus muscle; no difference in hot carcass weight, backfat thickness, and yield grade in beef steers (462)
Sheep	Moringa oleifera extract and *Bifidobacterium strain* of human origin *(B. pseudolongum INIA P2)*	Increased total phenolic content and antioxidant capacity of sheep milk cheese (463)
	Oligomeric Isomaltose and *Lactiplantibacillus plantarum* N-1	Improved production performance and meat quality of Hu sheep (464)
	*Enterococcus faecium* and Inulin	Increase in fiber digestibility and immunoglobulin concentration in blood of male sheep (465)
	*Bacillus cereus* and inulin, raffinose, and trehalose	Exhibited antibacterial effect against *E. coli* and *Salmonella* in sheep meat (mutton) (466)
	BALACTO^b^	Increased growth performance, feed conversion efficiency, and blood plasma total protein. Reduced the levels of urea, creatinine, cholesterol, triglyceride, and liver enzymes in lambs (467)
	Probiotics (live *Saccharomyces cerevisiae*) and prebiotics (mannan oligosaccharide plus b-glucans)	Improved dietary energetic efficiency in male lambs (249)
Swine	Probiotics and prebiotics^c^	Increased average daily gain, improved the quality of pork, and more favorable fatty acid composition of finishing pigs (468)
	Probiotics (*L. buchneri* NLRI-1201, L. plantarum NLRI-101, L. casei DK128) and prebiotic (fructo-oligosaccharide)	Modulated the microbiome in the proximal colon, oropharyngeal cavity and vaginal tract in female Korean Native Black pigs (469)
	Prebiotics (xylobiose, xylotriose, xylotetraose, xylopentose, xylohexaose, and xyloheptaose) and probiotics (*Lactobacillus plantarum* B90 and *S*. cerevisiae P11)	Improve meat quality, and gene expressions of muscle-fiber types (MyHCI, MyHCIIa, MyHCIIx, and MyHCIIb) and muscle growth and development (Myf5, Myf6, MyoD, and MyoG) genes in sow-offspring. Improve feed intake and promote the growth of piglets (470)
	*Bacillus coagulans* and Lactulose	Increased feed efficiency and showed greater resilience to acute immune stress in weaned piglets
Broilers	Probiotic (Primalac) and prebiotic (Fermacto)^d^	Improved feed conversion ratio, plasma lipid profile and antibody-mediated immunity of male broilers (471)
	Biomin Imbo	Reduced the number of *E. coli* and total coliform populations in the intestines of male chicks (472)
	Probiotics (Gut-pro^®^) and prebiotics (TGI^®^)	Increased carcass percentage; urea, uric acid and creatinine; and serum glutamic pyruvic transaminase in broiler chickens (473)
Fish	Biomin Imbo	Increased final body weight, feed conversion efficiency and survival rate in zebra fish (474)
	*Bacillus subtlis* and Fructooligosaccharide	Increased growth rate, feed efficiency ratio, non-specific immune responses and disease resistance in Juvenile large yellow croaker (475)
	Biomin Imbo	Increased growth performance, survival rate and feeding efficiency in rainbow trout (476)

^a^Symbioveba^®^ (probiotics [Lactobacillus and *Saccharomyces cerevisiae*), medicinal plants [*Taraxacum officinalis, Zingiber officinalis*], enzymes, plant extracts and water), obtained with the exclusive MESEN^®^ Patented process.

^b^BALACTO (Mannan oligosaccharides, β-GLUCAN, *Lactobacillus acidophilus*, and *Bacillus subtilis*); Bactizad for feed additives.

^c^Probiotics (*Lactobacillus plantarum, Bacillus subtilis*, and *Saccharomyces cerevisiae*) and prebiotics (glyconutrients composed of N-acetylglucosamine, D-xylose, and Fucose).

^d^Probiotics (*Lactobacillus casei, Lactobacillus acidophilus, Bifidobacterium bifidum* and *Enterococcus faecium*) and prebiotic (mannan-oligosaccharides derived from the outer cell wall of *Saccharomyces cerevisiae*).

### 4.5 Postbiotics

Postbiotics are also referred to as paraprobiotics, parapsychobiotics, ghost probiotics, metabiotics, tyndallized probiotics, and bacterial lysates. Postbiotics, as defined by the International Scientific Association of Probiotics and Prebiotics (ISAPP), are preparations of inanimate microorganisms and/or their components that provide health benefits to the host (252). A recent review updated this definition to include non-viable microbes or cell fragments, with or without metabolites, while emphasizing that purified metabolites alone do not qualify as postbiotics (253). Postbiotics have gained significant attention due to their enhanced safety, longer shelf life, and ease of production (254). They carry no risk of transferring antibiotic resistance genes and can be delivered to specific sites with encapsulation techniques, making them an attractive alternative in health and nutrition (255).

Several recent reviews have examined the effect of postbiotics use in livestock production (254, 256). Postbiotics improve growth performance (257, 258), increase nutrient digestibility and milk yield (259), improve gut health (260, 261), improve meat quality (262), anti-inflammatory and anti-oxidant properties (263, 264) in animals. For instance, Izuddin and colleagues reported that administering postbiotics from *L. plantarum* RG14, RG11 and TL1 affected antioxidant enzymes by increasing glutathione peroxidase in post-weaned lambs (264). Sample data on the impact of postbiotics feed additives on livestock health and performance is summarized in Table 9.

**Table 9:** Impact of postbiotic feed additives on livestock production and health.

**Livestock**	**Postbiotics**	**Research findings**
Cattle	Probisan-Ruminants-C^®^ (PC) and Probisan-Ruminants^®^ (PR)	Improved voluntary dry matter intake, improved apparent total tract digestibility of dry matter, organic matter, and neutral detergent fiber, increased colostral immunoglobulin concentrations, increased milk production, with elevated fat and protein yields and greater persistence in late gestation and early gestation dairy cow (259)
	*Saccharomyces cerevisiae* fermentation products	Increased the populations of lactate utilizing and fibrolytic bacteria (Ruminococcaceae and Lachnospiraceae), increased the numbers of hub taxa in non-sub acute ruminal acidosis (SARA) and SARA stages, prevented the fluctuations in the population of hub taxa that correlated positively with acetate concentration, and α- and β-diversity metrics in rumen liquid digesta of lactating dairy cows (477)
	*Saccharomyces cerevisiae* fermentation products	Improved milk yield and components, such as fat and protein, particularly during early lactation. Enhanced oxidative stability by reducing oxidative stress marker (malondialdehyde) and increased the levels of immunoglobulins and enhanced inflammatory responses in transition dairy cows (478)
	*Saccharomyces cerevisiae* fermentation products	Enhanced systemic immune responses, leading to increased interleukin-6 production in peripheral blood cells when stimulated with toll-like receptor ligands. Altered the expression of genes in the lung, favoring pathways associated with reduced inflammation and improved tissue repair. Enhanced plasminogen activity and glutathione metabolism in pre-weaning calves (479)
	Lactic acid bacteria (Kefir-derived *Lentilactobacillus* kefiri LK1 and normal raw milk- derived *Enterococcus faecium* EFM2)	Exhibited significant antimicrobial activity. However, kefir-derived postbiotics demonstrated a broader and more potent antimicrobial spectrum compared to those from raw milk *Enterococcus faecium* EFM2. Disrupted the biofilms of mastitis pathogens (*Staphylococcus aureus* and *E. coli, Enterococcus faecalis, Pseudomonas aeruginosa*) (480)
	*Saccharomyces cerevisiae* fermentation products	Decreased the levels of key pro-inflammatory cytokines, such as interleukin-6 (IL-6) and tumor necrosis factor-alpha in steers (481)
Goat	*Lactococcus lactis* strains (MK2/2, MK2/7, and MK2/8)	Inhibited harmful bacteria like enterococci and staphylococci, maintained good survival rates in goat milk yogurts, particularly strain MK2/8, which exhibited a high level of stability. Did not alter the pH of the yogurt, suggesting that their inclusion did not interfere with the fermentation process (482)
	Probisan Ruminants	Increased ruminal propionate concentration, enhanced fiber digestibility, reduced methane emissions, improved milk yield in lactating goats (483)
Sheep	Yeast postbiotics (rich in mannan-oligosaccharides and beta-glucans) and selenium-enriched yeast	Improved energy status, as indicated by lower β-hydroxybutyric acid levels, especially in the pre- and post-partum periods, increased superoxide dismutase activity and total antioxidant capacity, reduced protein carbonyls, suggesting reduced oxidative damage, suppresed pro-inflammatory cytokine genes (Chemokine [C-C motif] ligand 5, IL6, and Nuclear factor kappa B) in the ewes' immune cells (484)
	*Saccharomyces cerevisiae* var. Boulardii ATCC MYA-796	Enhanced the shelf life of lamb meat by reducing microbial growth, increasing antioxidant activity, and preserving the quality of the meat during storage, Improved the overall appearance, texture, and flavor of Lamb meat (485)
	*Lactobacillus paracasei* ATCC 55544	Reduced microbial growth on lamb meat (*E. coli*, and *Staphylococcus aureus* counts), extended the shelf life of lamb meat, preserved sensory attributes (meat odor, color, and appearance) (486)
	*Lactobacillus plantarum* (RG14, RG11 and TL1)	Postbiotic RG14 supplementation improved antioxidant status (higher serum and ruminal glutathione peroxidase levels and reduced serum thiobarbituric acid reactive substances), upregulated genes involved in ruminal barrier integrity including tight junction proteins (occludin, claudin-1 and claudin-4) in post-weaning lambs (264)
Horse	*Saccharomyces cerevisiae* fermentation product	Improved the stability and robustness of the equine gut microbiome under stress conditions in young horses (487)
	*Saccharomyces cerevisiae* fermentation product	Modulated pro-inflammatory and anti-inflammatory cytokine genes expresión in senior horses (488)
Swine	*Saccharomyces cerevisiae* fermentation media and extracellular metabolites	Improved growth performance and intestinal barrier function. Reduced diarrhea in piglets (489)
	*Saccharomyces* yeast postbiotics	Improved sow body condition and reduced the time between weaning and estrus, and enhanced the weight and growth rates of piglets at weaning. Increased average daily gain, feed intake, and feed efficiency and also improved fecal scores in nursery pigs. Increased growth performance during early stages of growing pigs (490)
	*Lactobacillus* postbiotics (*L. fermentum* and *L. delbrueckii*)	Modulate the composition of the mucosa-associated microbiota (increased *Prevotella stercorea* and *Dialister succinatiphilus)*, enhance the activity of pattern recognition receptors (PRRs) in the jejunal mucosa, and improve immune competence (491)
	*Saccharomyces* yeast postbiotics	Improved intestinal integrity (tended to increase the villus height and crypt depth ratio in the jejunum) and reduced oxidative damage (decreased the gene expression of serum and glucocorticoid-induced protein kinase 1). Reduced fecal score in the jejunal mucosa of young pigs (492)
	*Lactobacillus* fermentate	Improved intestinal health by enhancing the diversity and abundance of beneficial microbiota (*Lactobacillus salivarius* and *Propionibacterium acnes)*. Improved growth efficiency (increased average daily gain, increaed alpha diversity of jejunal mucosa) of nursery pigs challenged with F18+*E. coli* (493)
Fish	*Weissella cibaria* (CECT 30731 and CECT 30732)	Increased beneficial lactic acid bacteria but reduced total aerobic bacteria. Improved resistance to *Yersinia ruckeri* infection in rainbow trout (*Oncorhynchus mykiss*) (494)
	*Bacillus, Lactobacillus* and yeast (*Saccharomyces cerevisiae*)	Improved growth performance and survival rates. Increased antioxidant capacity, better immune responses, and enhanced gut health in juvenile oriental river prawn (*Macrobrachium nipponense*) (495)
	*Saccharomyces cerevisiae, Bacillus velezensis* and *Cetobacterium somerae*	Improved growth rate, nutrient absorption and enhanced immunity by downregulating the expression of pro-inflammatory cytokines (Nuclear Factor kappa-light-chain-enhancer of activated B cells p65 and Tumor Necrosis Factor-alpha) and upregulating anti-inflammatory cytokines (Interleukin 1 beta, interleukin 10, Transforming Growth Factor-beta) in common carp (*Cyprinus carpio*) (496)
Poultry	*Lactobacillus acidophilus*	Increased body weight, better feed conversion ratio, and improved immune responses. Reduced pathogen counts, such as total plate counts, coliforms, and increased beneficial bacteria like Lactobacillus. The gut morphology, particularly villus height and depth, as well as antioxidant levels in the jejunum, were significantly improved in broiler chickens (497)
	Culbac (a nonviable *Lactobacillus acidophilus* species fermentation producto)	Reduced the severity of necrotic enteritis caused by *C. perfringens*. Increased feed conversion ratio and improved overall health markers. Decreased mortality. Improved liver function and reduced bacterial counts. Improved immune response by increasing the inhibition of hemagglutination antibody titers for Newcastle disease virus vaccine in broiler chickens (257)
	*Lactiplantibacillus plantarum* (RG11, RI11, and RS5)	Improved body weight gain, feed conversion ratio, and mucin production. Improved gut integrity by upregulating the expression of intestinal mucins (Mucin 2), tight junction proteins like occludin, and secretory immunoglobulin A. Improved immune response by increasing the levels of glutathione peroxidase and superoxide dismutase. Reduced pathogenic bacteria like *E. coli* in broiler chickens (498)
	*Lactobacillus plantarum* RS5 fermented products	Increased egg production and improved feed conversion efficiency. Reduced oxidative stress by increasing antioxidant enzyme activities and decreasing malondialdehyde levels. Improved immune function and better overall health in layer hens under heat stress conditions (499)
	*Bacillus subtilis* ACCC 11025	Improved growth performance (bodyweight and feed efficiency). Increased the yield of breast and leg muscles, although the quality of these muscles remained unaffected by the supplementation. Beneficial effects on serum albumin and total protein levels. Reduced ammonia emissions from excreta. Modulated the microbiota in excreta, particularly increasing Lactobacillus counts while reducing *Salmonella* populations in broiler chicks (258)

The mode of action by which postbiotics exhibit their beneficial effects was recently reviewed (255). Postbiotics enhance gut health, nutrition, milk yield and composition, and immune response through various mechanisms. Postbiotics interfere with pathogen establishment and maintain the barrier function of the intestinal mucosa, thereby enhancing host–microbiome balance and contributing to a state of eubiosis. The effects of postbiotic treatments in reducing the severity of *E. coli* infections, improving gut health and growth performance in broilers and layers have been demonstrated (260, 261). The antimicrobial properties of postbiotics are mostly due to their metabolites (bacteriocins, short-chain fatty acids, etc.) which play significant roles in protecting against pathogen invasion and promoting gut health (265). Postbiotics modulate native probiotic strains, thereby maintaining intestinal microbiota and host homeostasis. Additionally, postbiotics can utilize bacterial extracellular vesicle secretion systems to mediate microbe–microbe communication (i.e. quorum sensing) and influence host signaling pathways (265). Postbiotics have longer shelf life, and are easy to produce (265).

Despite the numerous advantages associated with postbiotics, they also come with some limitations including lack of clear international regulatory standards, incomplete understanding of host-microorganism interactions which are responsible for producing specific postbiotics or a combination of postbiotics (266). Therefore, more research is needed to foster a more comprehensive approach and achieve harmonized regulatory frameworks.

### 4.6 Acidifiers

Acidifiers are compounds with acidic properties including organic and inorganic acids, and which demonstrate antimicrobial properties. Organic acids include benzoic, citric, formic, fumaric, lactic, and propionic acid or their salt counterparts (such as calcium, potassium, sodium formate, or sodium fumarate) (267, 268). Inorganic acids include hydrochloric, sulfuric, or phosphoric acids. Unlike inorganic acids, organic acids consist of one or several carboxyl (COOH) functional groups which play an important role in their activity. Some acidifiers can also form complexes with minerals including calcium (Ca^2+^) and zinc (Zn^2+^) cations that limit their absorption in the digestive tract. Acidifiers are generally recognized as safe agents and have been used in livestock diets and drinking water to improve their growth performance (269).

Organic acids have been found to improve gut health, nutrient digestibility and mineral utilization, inhibit inflammatory processes, and have antimicrobial effects (20, 270). The main mechanisms of the mode of action of acidifiers are: (i) reduction of the pH level in the gut which tend to reduce the numbers of pathogenic bacteria, (ii) direct killing of pathogenic bacteria by penetrating through their cell-wall, (iii) increase acid-tolerant beneficial species e.g., *Lactobacilllus* spp., (iv) increase nutrient digestibility by promoting retention of protein and dry matter, (v) improve absorption of mineral and use of phosphorous, and (vi) improve gut health via direct effects on epithelial cells (20). For decades, organic acids have been widely used as feed preservative due to their strong antibacterial and antifungal properties (271). The beneficial effects of organic acids are often enhanced when used as blends rather than as single products. Studies have demonstrated that organic acid blends improve feed conversion ratios in broiler chickens (272) and improve growth performance, serum antioxidant status, and intestinal health in weaned piglets (273), underscoring their potential as effective alternatives to antibiotics use in livestock production. Acidifiers positively influence intestinal microbiota, enhance immune response and growth performance, and reduce pathogen loads in livestock (20, 274). These beneficial effects of acidifiers in swine and poultry have been comprehensively reviewed (20, 275).

In spite of the demonstrated beneficial effects, the effectiveness of acidifiers can vary depending on factors such as the type of acid or form (acid, salt, coated or uncoated), chemical properties (pKa value, molecular weight, minimum inhibitory concentration of the acid), target microorganism, age of animal, diet composition and animals' health status and so on (20, 267, 270). For example, Ravindran and Kornegay reported that supplementing weaner pig diets with sulfuric acid posed a risk of reduced feed efficiency, likely due to alterations in electrolyte balance (276). Also, it was reported that although the inclusion of phosphoric acid effectively lowered pH, it did not improve nutrient utilization (277). Moreso, Walsh et al. (278) further reported that combining inorganic and organic acids could impair growth performance while the use of only the inorganic acid blend significantly improved growth performance compared to that of antibiotics or organic acid blends.

Acidifiers can be corrosive, potentially damaging equipment and posing handling issues for feed manufacturers (20). Acidifiers, when included at excessive levels, may reduce diet palatability and lead to decreased feed intake (267). For instance, studies by Shu et al. (279) and Mao et al. (280) reported that excessive consumption of benzoic acid can cause dysfunction and damage to the liver, spleen, and lungs, as well as alterations in gut morphology. In addition, acidifiers are very volatile, as a result, commercial acidifiers are often encapsulated with fatty acids or other materials to ensure controlled release in specific intestinal compartments (281). Although research evidence supports the use of organic acids for improving nursery pig growth, there is a lack of direct economic comparisons between commercial acidifier blends and conventional antimicrobials. This knowledge gap limits producers' ability to make well-informed, science-based decisions regarding their adoption in livestock production management (282).

### 4.7 Enzymes

Enzymes used as feed additives are biologically active proteins that promote the chemical breakdown of nutrients into smaller compounds for further digestion and absorption. Feed additive enzymes are derived from yeast, bacteria and fungi through fermentation (283). The most commonly used enzymes are phytase, carbohydrase, xylanase, α-galactosidase, β-mannanase, α-amylase, β-glucanase, proteases, lipases, and pectinase (284). Enzymes breakdown anti-nutritional factors found in plants such as phytic acid, non-starch polysaccharides, and cell-wall complex carbohydrates and improve nutrient utilization (284). Addition of enzymes to animal feed increase gut stability, enhance substrates for beneficial fermentation, and improve the ability of the intestines to fight against bacteria pathogens (285, 286). Yu et al. (286) found that the addition of carbohydrate enzymes and protease to the diet of finishing pigs improved the growth of beneficial bacteria (Actinobacteriota, Desulfobacterota, and Lactobacillus), reduced pathogenic Clostridium_sensu_stricto_1 and improved nutrient digestibility, average daily gain and growth performance of finishing pigs. Enzymes have also been used synergistically. A study discovered that the combined use of carbohydrases, phytase and acidifiers reduced *E. coli* counts and increased villus length in broiler chickens (287). Similarly, supplementation of multiparous early-lactation Holstein Friesian dairy cows' diet with fibrolytic (xylanase, cellulase and β-glucanase) and amylolytic (amylase) enzymes, enhanced α-amylase and xylanase activity levels in rumen fluid, improved milk production and feed efficiency (288).

The possible mechanisms of action of in-feed enzymes include the breakdown of plants anti-nutritional substances (e.g., phytic acid and non-starch polysaccharide); improve nutrient availability by degrading the cell-wall of complex polysaccharides; improve nutrient digestion of young animals having less developed digestive system; reduce feed costs and improve feed efficiency. Furthermore, in-feed enzymes exhibit antimicrobial effects by directly hydrolyzing bacterial cell walls or compromising the integrity of glycocalyx's (289).

The main caveats associated with the use of in-feed enzymes include the lack of proper understanding on inclusion rates, low enzyme activity, the dependence of enzyme function on diet composition, ambient temperature and pH, their susceptibility to inactivation by the high levels of gut acidity and low production and quality control standards (290). The beneficial effect of enzyme inclusion is sometimes inconsistent because of different diet composition, animal genetic variations and type of enzymes used (289). The effects of in-feed enzymes are variable on animal growth performance. Another limitation is the concern about the ability of hydrolytic enzymes to survive processing temperature and the animal digestive tract.

### 4.8 Clay minerals

Clay minerals (e.g., bentonite, sericite, Biotite V, zeolite, kaolin etc.) have stratified structures and as a result possess great adsorption capacity enabling them to bind aflatoxins, heavy metals, enterotoxins, pathogens, and plant metabolites (248). Clay minerals such as bentonite, zeolite and montmorillonite are characterized by their negatively charged and high surface areas, swelling ability, pore volume, and high cation exchange capacity. Moreover, mineral adsorbents such as hydrated sodium calcium aluminosilicate can bind or adsorb mycotoxins to their interlayer spaces, external surface and edges. These properties cause reductions in microbial metabolites, toxins, and enzymes in the intestine; thereby preventing irritation and damage and improving performance and morphological characteristics of the intestinal mucosa (291). Therefore, there is growing interest in the use of clay minerals due to their beneficial characteristics, absence of primary toxicity and ability to reduce animal diseases and improve animal production and safety of animal products.

The feeding of clay minerals to livestock has resulted to reduction in diarrhea, better feed conversion ratio, and improved health (292, 293). Clay minerals have been reported to fully or partly mitigate the toxic effects of mycotoxins in farm animals fed with contaminated diets (294, 295). The antibacterial mechanism of medicinal clays involves the release of soluble ferrous iron and aluminum ions (Fe^2+^ and Al^3+^) that attack cellular systems in pathogens (296). The antibacterial effectiveness of clays is influenced by their mineralogical variables, such as pH and Eh (redox potential) conditions (297). Clay minerals have also been used as drug carriers for antibiotics, demonstrating their potential in healthcare applications (298). In animals, the inclusion of 5 g/kg hydrated aluminosilicate into broiler diets significantly increased body weight gain of broilers at 1 and 3 weeks of age and increased the activity of serum amylase and lactate dehydrogenase (299).

The use of clay minerals has several limitations including incomplete understanding of the bacteriostatic mode of action (293). More so, when clay minerals are added to feed at high concentrations, they can bind to other nutrients like vitamins and trace elements, causing nutritional imbalance for animals (293). There can be negative effects induced in farm animals due to interaction with veterinary substances and micronutrients in feed as well as *in vitro* and *in vivo* toxicity of natural and modified mineral adsorbents. Davis et al. (300) reported that high adsorption capacity of thermally processed clays could lead to unintended adsorption of essential vitamins and trace minerals along with targeted biotoxins. Clay minerals utilization can lead to contamination of livestock feed (301).

### 4.9 Heavy metals and rare earth elements

Heavy metals (copper, zinc, iron, selenium, manganese, etc.) are known as trace minerals in animal nutrition and are mainly used to improve animal health and physiological functions (302). Rare earth elements are a group of 17 elements, including 14 chemical elements from group 3A of the periodic table called lanthanoids (atomic numbers 58–71) and three members in group 3B, namely scandium (Sc; atomic number 21), yttrium (Y; atomic number 39) and lanthanum (La; atomic number 57) (303).

Heavy metals are commonly used as in-feed supplements and they play significant roles in improving digestibility, physiological, and biosynthetic processes (285, 304). Zinc is known to play essential roles in cell proliferation, immune response, reproduction, gene regulation, and defense against oxidative stress and damage (305–307). For example, there was significant improvement in body weight gain of broilers supplemented with 80 mg/kg of zinc (308). Yusof et al. (309) observed that supplementation of broiler diet with 100 mg/kg of zinc oxide nanoparticles increased body weight gain and reduced pathogenic bacteria count (*Enterococcus* spp. and *E. coli*) without affecting the beneficial bacteria in the GIT. The growth-promoting effects of copper and the mechanisms of action have been reviewed recently (310). Divalent copper(II) sulfate (CuSO_4_) and monovalent copper oxide (Cu_2_O) are commonly used as growth promoter in pig (304, 311) and poultry (312).

A plethora of investigations have reported on the use of rare earth elements as alternative to antibiotics use in livestock production (303, 313, 314). For example, Xu et al. provide insights into the antibacterial activity of specific rare-earth ions (Yb^3+^, Gd^3+^, Sm^3+^, Tb^3+^, and La^3+^) against *S. aureus* and *Pseudomonas aeruginosa*, demonstrating their efficacy against both planktonic and biofilm-forming bacteria. The efficacy of the rare earth elements were compared to that of traditional agents like Cu^2+^, Ag^+^, and the antibiotic norfloxacin. However, while higher concentrations of rare-earth ions were required to achieve similar antibacterial effects, they exhibited significantly lower cytotoxicity toward mammalian cells. Notably, Gd^3+^ demonstrated potent bactericidal activity against both planktonic and biofilm bacteria with the lowest cytotoxicity, and all tested rare-earth ions showed excellent antifungal activity against *Candida albicans*, suggesting their potential as safer alternatives for developing novel antimicrobial agents (315).

Despite the benefits of using heavy metals and rare earth elements in livestock production, their application still faces certain limitations. For example, it has been observed that dietary supplementation of animal diets with heavy metals (e.g., Cu and Zn) can cause an increase in antibiotic resistance through co-selection and mobilization of ARGs (311). ARGs and metal resistance genes (MRGs) are genetically linked (i.e., co-resistance) if the resistance mechanism is the same with the resistance to both metals and antibiotics (i.e. cross-resistance), or if the expression of resistance systems to both metals and antibiotics is controlled by the same factor (i.e., co-regulation), these can lead to occurrence of co-selection (311, 316). Furthermore, inclusion of high dose of heavy metals can affect the absorption of essential trace elements and cause heavy metal residues, and it can be detrimental to the environment due to their accumulation in soil and surface water (308, 317). Likewise, excess dose of heavy metals could lead to immunosuppression, impaired health and negative effects on reproductive performance in livestock (318).

Moreover, the effectiveness of rare earth elements can vary depending on several factors, including bioavailability, the specific rare earth element compound used, the animal species, and the dosage (319). As cations, rare earth elements can interact with anions, potentially affecting their absorption and biological activity (319). The form in which the rare earth element is administered can influence its efficacy. For example, He et al. (319) reported that broilers supplemented with rare earth element citrate (an organic form) at a low dose of 70 mg/kg showed significantly improved growth performance, whereas the chloride form had no effect on their growth performance. Likewise, differences in animal species affect the effectiveness of rare earth elements (320). Therefore, further research is required to elucidate the underlying mechanisms of the effects of rare earth elements and to establish optimal use in livestock production.

## 5 Economics and the One Health implication of the use of alternatives to antibiotics in animal production

The use of effective and eco-responsible alternatives to antibiotics in agriculture will not only have a positive impact on industry and animals, but also on the environment and society in general. The economic value of livestock production without the use of antimicrobials is influenced by many factors including input and output prices, risk aversion, and profit variance. Other situations and factors include farmers' perceptions of costs and risks, and limited access to cheap sources of inputs (321, 322). Therefore, understanding these factors can help identify pathways to reduce antimicrobial use. However, while reducing antimicrobial use is crucial, there's no global consensus on acceptable levels, and a complete ban could have adverse effects on animal health and welfare (321). Many cost-effective antimicrobial alternatives are still experimental, requiring further research. Despite the lack of consensus, reducing antimicrobial use is widely agreed upon, and this serves as a basis for developing effective policies (322). Utilizing interdisciplinary systemic approaches facilitates the development of AMR policies and strategies that are feasible across technical, political, economic, and behavioral dimensions. This approach enables the identification of various key factors, including all factors influencing antimicrobial use in livestock production, the power dynamics between these factors, appropriate regulatory frameworks and interventions, optimal behavioral change strategies, and the responsible entities for implementation (323). Additionally, it allows for the cost-effective assessment of intervention combinations. However, AMR policies and strategies are often explored within different disciplines, lacking a holistic and systemic perspective. Therefore, advocating for more interdisciplinary collaboration is crucial to addressing the use of antimicrobials in livestock production and the emergence of AMR effectively, and further research opportunities in this area is warranted.

From an economic standpoint, antimicrobial use in livestock production generates negative externalities (e.g., development of AMR and the impact of AMR on the environment and society), and assessing the total economic value of antimicrobial use should consider economic losses due to the impact of AMR in the One Health continuum. This implies that the economic impact of AMR extends beyond increased healthcare costs and treatment failures in humans to include interruptions in agricultural productivity and international trade. For example, zoonotic disease outbreaks caused by multidrug-resistant pathogens, such as *Salmonella* and avian influenza have resulted to economic losses to the tune of billions of US dollars due to culling, decreased consumer confidence in livestock-derived products and trade restrictions (324). In dairy production, multidrug-resistant *S. aureus* strains causing mastitis significantly impacts milk production, leading to economic losses and increased dependance on antimicrobials for treatment (325). Also, many studies have reported that many commonly used intramammary antibiotic formulations contain critically important antimicrobial classes, such as aminoglycosides and cephalosporins, which are crucial for human medicine (326). The frequent use of these antibiotics not only accelerates resistance development but also increases the risk of economic loses and the presence of antimicrobial residues in dairy products, further complicating efforts to mitigate AMR.

Despite risks posed by AMR, many farmers, veterinarians and pharmaceutical companies continue to consider antimicrobials as essential components of livestock production, by prioritizing production efficiency and economic gains over the responsible use of antimicrobials. Recent surveys of dairy farmers in Scotland and Switzerland have identified key demographic and behavioral predictors of antimicrobial use trends, with younger farmers and those managing larger herds being more likely to engage in high antimicrobial usage as well as the tendency of farmers to rely on peer recommendations rather than veterinary guidance or self-prescribe antibiotics, which further complicates efforts to mitigate AMR (325, 326). Moreover, veterinarians as key decision-makers in antimicrobial use also face significant challenges in promoting antimicrobial stewardship as they are often pressured to prescribe antimicrobials based on farmers' preferences rather than scientific evidence (326). Despite the threat of AMR, a 2019 investigation by the New York Times found that Elanco, an animal pharma company, created a brochure that encouraged farmers to treat whole herds of pigs with antibiotics to prevent disease (327).

The challenges of limiting antimicrobial use in livestock production are particularly obvious in regions with limited veterinary oversight and weak regulatory frameworks (328). Therefore, addressing these issues requires the implementation of sustainable practices including the use of antimicrobial alternatives, enhanced antibiotic stewardship, strengthened interdisciplinary collaboration and emphasizing the critical need for a One Health approach by recognizing the interconnectedness of human, animal, and environmental health in safeguarding global public health and mitigating AMR (329). Such an approach is valuable for developing effective strategies for combating this common foe (AMR), including strengthening biosecurity measures, education of farmers on the implication of AMR and emphasizing responsible antimicrobial usage for curative purpose only, while eliminating use in growth promotion.

## 6 Research gaps, future perspectives and conclusion

Finding functional and more sustainable alternatives to antibiotics as growth promoters is expected to increase the wide range of different alternatives to be included in further research. Although only few available vaccines can effectively control disease occurrence and eliminate the therapeutic use of antimicrobials, and while few/no alternatives have emerged to replace antibiotics as growth promoters in livestock production, the options discussed in previous sections hold significant value that can be integrated into practical “no antimicrobial use as growth promoters” in livestock production programs. This involves strengthening collaborations with local farmers and animal husbandry practitioners to ensure real-world applicability of research findings; engaging stakeholders through international conferences and agricultural forums to raise awareness and foster interdisciplinary dialogue; promoting public-private partnerships and community-based projects to secure funding and logistical support for field trials; integrating knowledge-sharing platforms to disseminate best practices and align local efforts with global One Health goals. However, the use of most of these alternatives is still limited due to reliability, species-specificity, costs and complexity of production.

Currently, the mechanism of action of most antimicrobial alternatives are not known. For example, the intestinal physiology and microbiota of different breeds may affect the effectiveness of an alternative, be it probiotics or other alternatives presented above. It should be noted that the GIT consist of a wide array of nutritional and physicochemical environments which may impact the colonization and survival of supplemental probiotics. Moreover, there is a lack of understanding of the nature, function, and role of biofilm-forming microbial communities in pathophysiology and animal health. Considering that biofilm growth is common in GIT diseases, developing alternatives to antibiotics should take into consideration and explore how antibiotic alternatives in animal feed can affect biofilm growth and survival. The synergistic effect of probiotics and prebiotics on host physiology, the immune response and optimal ratios and rate of inclusion in livestock feed deserves scrutiny. The interactions between the microbiota, host, and feed components are not fully understood, which hampers their widespread use. Additionally, the potential for resistance development and co- and cross-resistance with antibiotics is a concern (20). To achieve maximum results, it is important to determine the exact dosage for each breed and species. In addition, microbial alternatives to AGPs should be broadly applicable, have low adverse effect levels, generally recognized as safe to livestock and the environment, and pose no threat to AMR development.

Understanding host-pathogen interactions and immune response to pathogens will improve the development of precise and personalized immunotherapies. Likewise, developing effective combination of immunotherapies will lead to synergistic effects and enhanced outcomes for disease prevention and treatment, following thorough evaluation and optimization of dosages, timing, and interactions between the components. For example, the use of AMPs shows promising positive impact on the host immune systems, however, more studies are needed to improve their use in animal production, and to properly understand their synergistic effect with other AGPs. The use of nanotechnology-based approaches, such as nanoparticle vaccines, can enhance the efficacy of vaccines and enable targeted delivery of immunotherapeutic agents to the site of infection, thereby enhancing their effectiveness and minimizing their side effects. Combination of different omics technologies, such as genomics, epigenomics, transcriptomics, etc. could provide important insights into the host immune response to livestock disease pathogens and the development of effective immunotherapies.

Antimicrobial alternatives delivered nutritionally (feed and water) pose numerous challenges needing concerted efforts to address them. Regarding phytochemicals, there is lack of understanding of the biochemical route or mechanism of action by herbs and spices, which calls for further research to understand their mechanisms of action and their rate of inclusion in livestock diets. Offensive characteristics (bad odors, variations in product compositions, high volatility, etc.) limits the application of essential oils in animal nutrition. Therefore, deeper understanding on their mechanism of action, optimal dosage and duration regime, and mode of administration is warranted to improve their efficiency and safety. Further research is also needed to optimize the delivery of essential oils into the lower GIT using microencapsulation and nanotechnology, understand the potential synergistic effects of combining different essential oils and other compounds, and dissect the mechanisms underlying the functions of essential oils using high-throughput systems technologies (19). Additionally, the potential for essential oils to interact with feed and the gut ecosystem of animals requires further investigation (330). Deep knowledge of organic acids and in-feed enzymes mode of action and potential interactions with other feed additives or medications is needed to fully understand their application as alternatives to AGPs in livestock production. Similarly, further studies are needed to certify the effectiveness and safety of utilizing clay minerals effectively in livestock nutrition while minimizing potential adverse effects, and to elucidate the safe use of heavy metals in animal nutrition.

While the antimicrobial alternatives discussed above present potential as replacements for antimicrobials in livestock production; perhaps, there should be a rethink of the goals for the search of effective AGP alternatives. For example, instead of searching for products with similar mode of action to antibiotics, the focus should be directed at products with ability to improve productivity and enhance animal health with the consequence of reduced need for therapeutic and sub-therapeutic antibiotics use. To focus in this direction, the mode of action of AGPs in health and production enhancement must be understood and management practices tailored to keep-away disease causing pathogens. However, AGP function is more complex than merely modulating microbial populations and functions, and their mode of action may be through direct or indirect modulation of the host immune system and consequently the immune response. Therefore, the key to finding new effective AGP alternatives lies in knowledge of the mechanisms associated with their action, the realization that no alternative can elicit the same mechanisms of action ascribed to AGPs and a focus on a combination of strategies (e.g., combine two or more AGP alternatives, AGP alternative and management practices, etc.) to achieve desired improvements without AGP. Moreover, a One Health approach should be applied to the search for AGP alternatives, with the aim to develop strategies that could simultaneously address animal health, human health, and the overall health of the ecosystem. A comprehensive understanding of these future perspectives will lead to significant advancements in the search for AGP alternatives for addressing the prevention, treatment, and control of livestock diseases, for the benefit of the livestock industry and animal and human welfare.

In conclusion, there are currently few or no alternative products that could completely replace antibiotics as a growth promoter in livestock production. However, the synergistic use of different alternatives could yield optimum breakthroughs in this field. While utilizing a combination of alternatives to antibiotics appears to be a promising strategy to combat drug resistance, it is not without its drawbacks. As we continue to strive toward reducing the use of antibiotics as growth promoters, it is crucial to give special attention to sustainable manufacturing, environmental impact, the likelihood of resistance development, the genetics of resistance evolution, and the risk of antibiotic cross-resistance. These factors should be carefully considered during the development and regulation of alternatives to antibiotics in livestock production.
